# Understanding the treatment benefit of hyperimmune anti-influenza intravenous immunoglobulin (Flu-IVIG) for severe human influenza

**DOI:** 10.1172/jci.insight.167464

**Published:** 2023-07-24

**Authors:** Hillary A. Vanderven, Deborah N. Wentworth, Win Min Han, Heidi Peck, Ian G. Barr, Richard T. Davey, John H. Beigel, Dominic E. Dwyer, Mamta K. Jain, Brian Angus, Christian T. Brandt, Analia Mykietiuk, Matthew G. Law, James D. Neaton, Stephen J. Kent, for the INSIGHT FLU-IVIG Study Group

**Affiliations:** 1 Biomedicine, College of Public Health, Medical and Veterinary Sciences, and; 2 Australian Institute of Tropical Health and Medicine, James Cook University, Douglas, Queensland, Australia.; 3 Department of Microbiology and Immunology, Peter Doherty Institute for Infection and Immunity, University of Melbourne, Parkville, Victoria, Australia.; 4 Divison of Biostatistics, School of Public Health, University of Minnesota, Minneapolis, Minnesota, USA.; 5 Kirby Institute, University of New South Wales, Sydney, New South Wales, Australia..; 6 WHO Collaborating Centre for Reference and Research on Influenza at the Peter Doherty Institute of Infection and Immunity, Melbourne, Victoria, Australia.; 7 National Institute of Allergy and Infectious Disease (NIAID), Bethesda, Maryland, USA.; 8 New South Wales Health Pathology-Institute of Clinical Pathology and Medical Research, Westmead Hospital, Westmead, Australia.; 9 UT Southwestern Medical Center, Dallas, Texas, USA.; 10 Nuffield Department of Medicine, Oxford University, Oxford, United Kingdom.; 11 Department of Infectious Diseases, Zealand University Hospital Roskilde, Denmark.; 12 Instituto Médico Platense, Buenos Aires, Argentina.; 13 Melbourne Sexual Health Centre and Department of Infectious Diseases, Alfred Health, Central Clinical School, Monash University, Carlton, Victoria, Australia.; 14 The INSIGHT FLU-IVIG Study Group is detailed in Supplemental Acknowledgments.

**Keywords:** Immunology, Infectious disease, Adaptive immunity, Influenza, NK cells

## Abstract

**BACKGROUND:**

Antibody-based therapies for respiratory viruses are of increasing importance. The INSIGHT 006 trial administered anti-influenza hyperimmune intravenous immunoglobulin (Flu-IVIG) to patients hospitalized with influenza. Flu-IVIG treatment improved outcomes in patients with influenza B but showed no benefit for influenza A.

**METHODS:**

To probe potential mechanisms of Flu-IVIG utility, sera collected from patients hospitalized with influenza A or B viruses (IAV or IBV) were analyzed for antibody isotype/subclass and Fcγ receptor (FcγR) binding by ELISA, bead-based multiplex, and NK cell activation assays.

**RESULTS:**

Influenza-specific FcγR-binding antibodies were elevated in Flu-IVIG–infused IBV- and IAV-infected patients. In IBV-infected participants (*n* = 62), increased IgG3 and FcγR binding were associated with more favorable outcomes. Flu-IVIG therapy also improved the odds of a more favorable outcome in patients with low levels of anti-IBV Fc-functional antibody. Higher FcγR-binding antibody was associated with less favorable outcomes in IAV-infected patients (*n* = 50), and Flu-IVIG worsened the odds of a favorable outcome in participants with low levels of anti-IAV Fc-functional antibody.

**CONCLUSION:**

These detailed serological analyses provide insights into antibody features and mechanisms required for a successful humoral response against influenza, suggesting that IBV-specific, but not IAV-specific, antibodies with Fc-mediated functions may assist in improving influenza outcome. This work will inform development of improved influenza immunotherapies.

**TRIAL REGISTRATION:**

ClinicalTrials.gov NCT02287467.

**FUNDING:**

Funding for this research was provided by subcontract 13XS134 under Leidos Biomedical Research Prime Contract HHSN261200800001E and HHSN261201500003I, NCI/NIAID.

## Introduction

The need for antibody-based prophylactics and therapeutics targeting respiratory viruses has become increasingly urgent. Previous studies and meta-analyses suggested passive infusion with convalescent plasma or hyperimmune anti-influenza intravenous immunoglobulin (Flu-IVIG) may decrease mortality during severe influenza A virus (IAV) infections with the 1918 and 2009 pandemic A/H1N1 strains (1–3). However, some of these studies lacked the design rigor and laboratory analyses to definitively assess the utility of this strategy. Over the past decade, antibody-based options for the treatment and prevention of severe human influenza, including both polyclonal and monoclonal antibodies (mAbs), have been widely tested in preclinical animal models and clinical trials (4–21). Despite being deemed safe and well tolerated, clinical trials of antibody-based therapeutics for IAV have generally shown minimal or no impact on measures of clinical outcome (4, 6, 8, 11, 17). To date, there are no approved antibody-based therapies to treat human influenza (17).

A recent (*n* = 329) randomized, double-blind, placebo-controlled, phase III clinical trial (INSIGHT 006; ClinicalTrials.gov NCT02287467) revealed that the robust rise in hemagglutination inhibition (HAI) titers following Flu-IVIG treatment did not result in any clinical benefit for IAV-infected participants (8). In contrast to the lack of efficacy for IAV, a beneficial effect of Flu-IVIG in influenza B virus–infected (IBV-infected) patients was evident (8). Interestingly, IBV antibodies in the Flu-IVIG preparations had a higher affinity than IAV antibodies, leading to slower dissociation rates of antigen-antibody complexes or immune complexes (8). The functionality of immune complexes is largely driven by interactions with Fcγ receptors (FcγRs) expressed on immune effector cells, but the underlying mechanism of Flu-IVIG protection in severe influenza B is unknown.

HAI antibodies prevent influenza virus attachment and entry into host cells by binding to epitopes adjacent to the receptor binding site of hemagglutinin (HA). However, the epitopes targeted by HAI antibodies typically accumulate mutations and glycosylation that lead to a narrow strain specificity (22–24). Since Flu-IVIG is manufactured months in advance of clinical use, it may contain limited or suboptimal levels of HAI antibody against antigenically drifted, pandemic or emerging zoonotic influenza viruses. Antibodies targeting the envelope protein neuraminidase (NA) can also block progeny virions from budding out of influenza-infected cells, reduce disease severity, and perform Fc effector functions (25, 26). We have previously shown that Flu-IVIG preparations contain broadly reactive anti-HA and -NA antibodies capable of mediating antibody-dependent cellular cytotoxicity (ADCC) and these antibodies were boosted after Flu-IVIG infusion (27).

Antibodies with Fc-mediated effector functions can target more conserved epitopes than HAI antibodies and are increasingly recognized as potential mediators of influenza immunity (28–37). Immune effector cells, including natural killer (NK) cells and macrophages, express FcγRs that can interact with the Fc domain of immunoglobulin G (IgG) (38, 39). Multimeric engagement (or cross-linking) of FcγRIIIa by IgG bound to virally infected cells stimulates the release of cytokines and cytotoxic granules from NK cells, which can help to clear infected cells (29, 30, 40). Furthermore, cross-linking of FcγRIIa on phagocytes by multiple IgG Fc domains leads to phagocytosis of virions and infected cells (41). Passive infusion studies in mice have shown that Fc-mediated effector functions can be a key component of protection against lethal IAV and IBV infections (25, 28, 42). It is plausible that Fc functions like ADCC represent potential mechanisms of protective immunity in IBV-infected humans following Flu-IVIG infusion.

Herein, we aim to determine whether influenza-specific Fc-functional antibodies were increased following Flu-IVIG treatment compared with placebo in IBV-infected patients, with IAV-infected participants included as a comparator group. The secondary aim of this study was to examine associations between influenza-specific antibodies and ordinal clinical outcomes of infection. These detailed mechanistic analyses of the INSIGHT 006 trial provide valuable information regarding potential antibody features and mechanisms required for a successful humoral response against influenza viruses, which will inform further research and development of improved immunotherapies for severe human influenza.

## Results

### Flu-IVIG provides a treatment benefit for influenza B but not influenza A.

The INSIGHT 006 double-blind, randomized, placebo-controlled phase III clinical trial (NCT02287467) enrolled 329 IAV- and IBV-infected patients from 2014 to 2018 (8). Of the 308 patients included in the primary analysis (mean age 57 years, 55% female), 156 received 500 mL of the Flu-IVIG infusion (0.25 g/kg to a maximum of 24.75 g) plus standard care (Flu-IVIG group) and 152 received 500 mL of saline as placebo plus standard care (placebo group) (8). A total of 224 out of the 308 patients (72.7%) were infected with IAVs (A/H1N1, *n* = 73; A/H3N2, *n* = 137; and A/subtype unknown, *n* = 14) and 84 out of the 308 patients (27.3%) were infected with IBVs (B/Yamagata, *n* = 64; B/Victoria, *n* = 13; and B/lineage unknown, *n* = 7) (8). The primary endpoint of the INSIGHT 006 trial was to determine whether there was any clinical benefit of Flu-IVIG infusion in patients hospitalized with severe influenza. Subgroup analyses revealed that Flu-IVIG infusion provided a clinical benefit in the patients infected with IBV but not IAV. This finding, along with the observed higher affinity of IBV antibodies in Flu-IVIG, motivated further serological analyses to understand the protective mechanisms of Flu-IVIG in patients with severe influenza.

**Figure 1:**
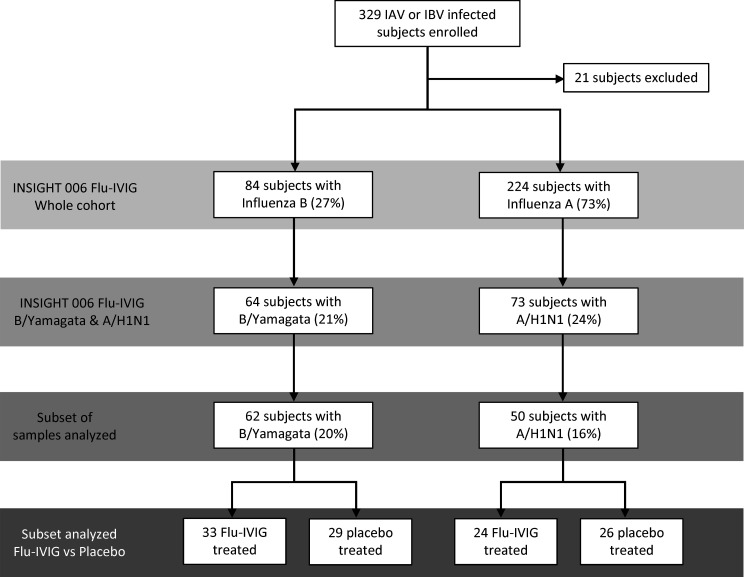
Flow diagram depicting the subset of patients from the INSIGHT 006 Flu-IVIG clinical trial who underwent detailed serological analyses. Of the 308 participants included in the primary analysis, 84 (27%) had influenza B and 224 (73%) had influenza A. Of the 84 participants with influenza B, 64 patients (21% of total participants) were infected with a B/Yamagata lineage influenza virus. Of the 224 participants with influenza A, 73 patients (24% of total participants) were infected with an A/H1N1 influenza virus. Based on sample availability, serological analyses were performed with 62 sera samples from B/Yamagata-infected patients (20% of total participants) and 50 sera samples from A/H1N1-infected patients (16% of total participants). In the analyzed B/Yamagata-infected participants, 29 were infused with placebo and 33 infused with Flu-IVIG. In the analyzed A/H1N1-infected participants, 26 received placebo and 24 received Flu-IVIG.

For this study, we analyzed a subset of 112 participants from the recently published INSIGHT 006 Flu-IVIG trial, who were infected with either A/H1N1 or B/Yamagata and had sera available for study. These patient groups were selected for further serological analyses due to B/Yamagata being the more common IBV infection (64 cases of B/Yamagata vs. 13 cases of B/Victoria) and A/H1N1 influenza virus having higher antigenic stability relative to A/H3N2 viruses over the 5-year period of the trial. Paired preinfusion (day 0, d0) and day 1 (d1) postinfusion sera from 62 B/Yamagata IBV-infected patients (out of 64 enrolled) and 50 A/H1N1 IAV-infected patients (out of 73 enrolled) were tested (*n* = 112), not all sera could be included due to limited sample availability. The B/Yamagata-infected group includes 33 patients who received Flu-IVIG and 29 patients who received the placebo infusion. The A/H1N1-infected group is comprised of 24 patients who received the Flu-IVIG infusion and 26 patients who received placebo (Figure 1). The B/Yamagata-infected group was older (median = 59 years, IQR = 44–64) than the A/H1N1-infected group (median = 52 years, IQR = 45–62) and were more likely to be enrolled in the last 1.5 years of the study between October 2016 and May 2018 (Table 1). Clinical outcomes were assessed on day 3, day 5, and day 7 after infusion using a 6-category ordinal outcome with the following mutually exclusive categories: (i) death, (ii) in intensive care, (iii) hospitalized but requiring supplemental oxygen, (iv) hospitalized and not requiring supplemental oxygen, (v) discharged from hospital but unable to resume normal activities, and (vi) discharged from hospital and able to resume normal activities.

**Figure 2:**
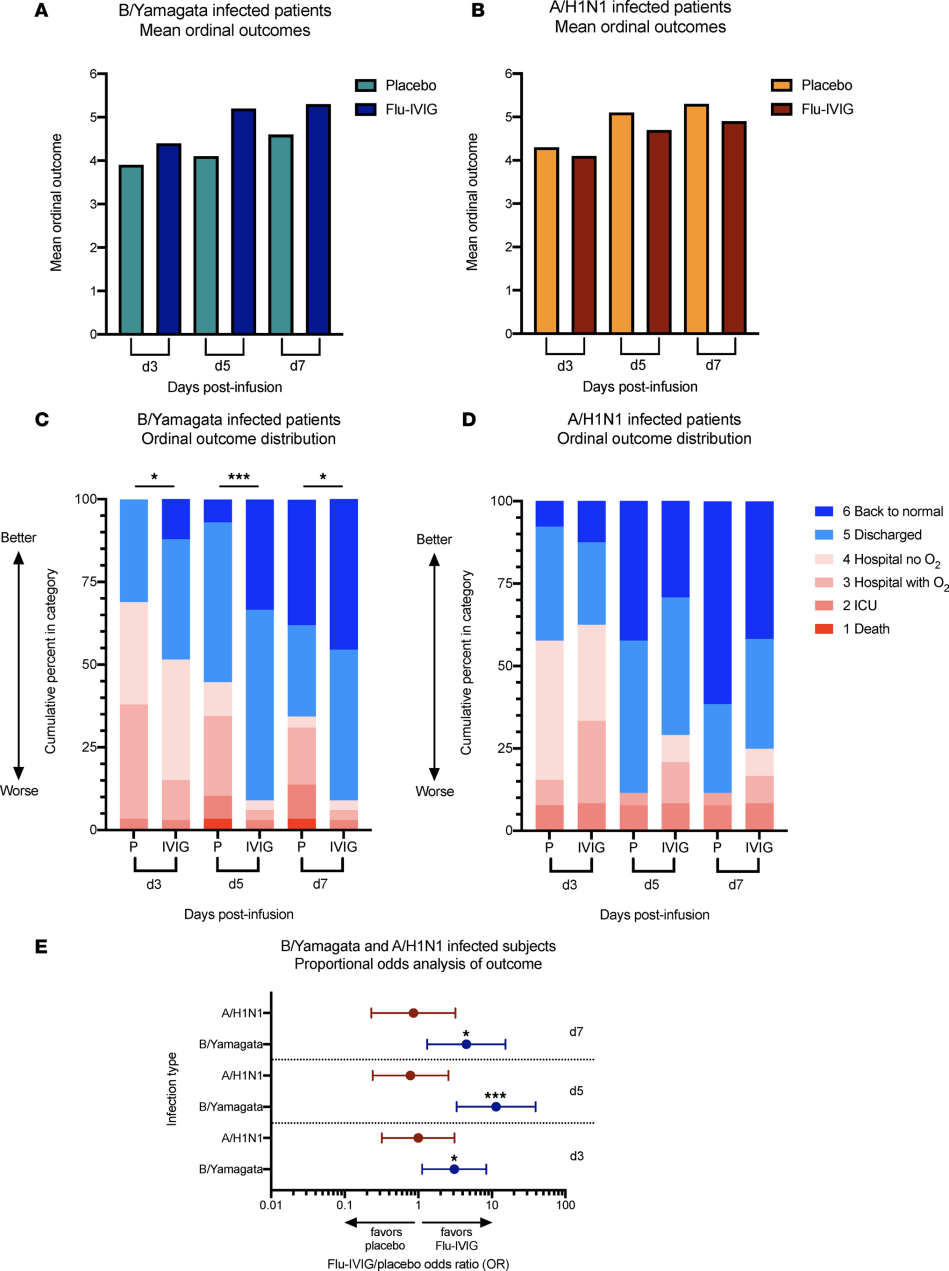
Treatment with Flu-IVIG improves the odds of a more favorable clinical outcome in patients hospitalized with severe B/Yamagata influenza but not A/H1N1 influenza. Mean ordinal outcomes (**A** and **B**) and ordinal outcome distributions (**C** and **D**) on day 3 (d3), d5, and d7 after infusion are shown for the Flu-IVIG– and placebo-infused patients infected with severe B/Yamagata influenza (Flu-IVIG *n* = 33 and placebo *n* = 29; **A** and **C**) and A/H1N1 influenza (Flu-IVIG *n* = 24 and placebo *n* = 26; **B** and **D**). The Flu-IVIG/placebo odds ratios (ORs) with 95% confidence intervals for the B/Yamagata- and A/H1N1-infected participants were calculated on d3, d5, and d7 after infusion (**E**). A proportional odds model with adjustment for the patient’s baseline clinical status was used to compare the Flu-IVIG– and placebo-treated groups. *P <0.05; ***P <0.001.

In the subset of 112 participants analyzed from the INSIGHT 006 study, the B/Yamagata patients who received Flu-IVIG had significantly better odds of a more favorable clinical outcome at all postinfusion time points compared with patients who received placebo (d3 odds ratio [OR] = 3.1, 95% CI = 1.1–8.4; d5 OR = 11.4, 95% CI = 3.3–9.3; d7 OR = 4.5, 95% CI = 1.3–15.4; Figure 2). There was no treatment effect of Flu-IVIG in the A/H1N1-infected participants at any of the time points tested (d3 OR = 1.0, 95% CI = 0.3–3.1; d5 OR = 0.8, 95% CI = 0.2–2.6; d7 OR = 0.9, 95% CI = 0.2–3.2; Figure 2). These results are consistent with findings from the whole INSIGHT 006 cohort, where patients with influenza B received a treatment benefit from Flu-IVIG, but patients with influenza A did not.

### Treatment with Flu-IVIG boosts HAI in patients with influenza A and B.

**Figure 3:**
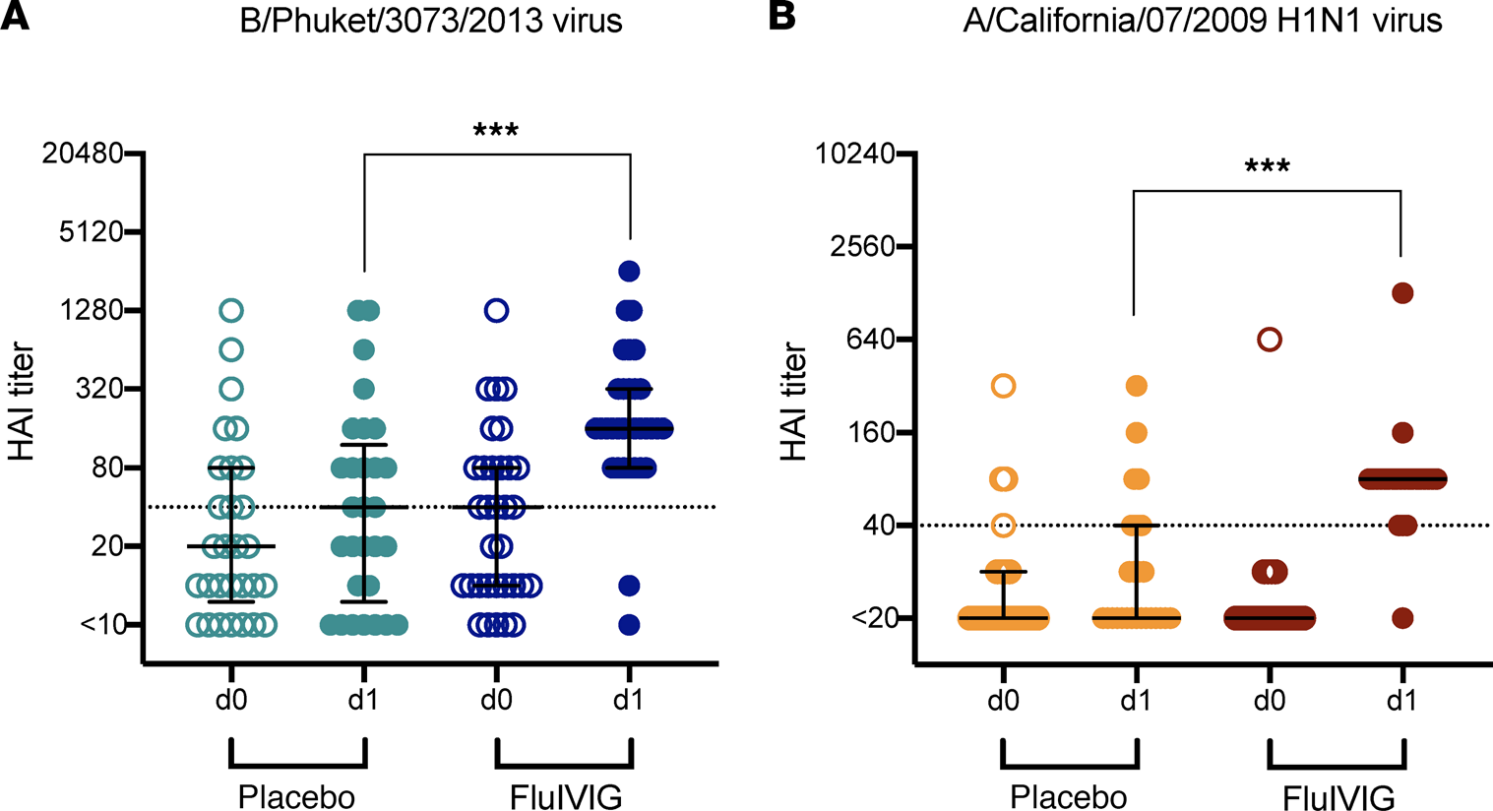
Hemagglutination inhibition (HAI) titers 1 day after infusion with influenza-specific hyperimmune immunoglobulin (Flu-IVIG) in patients hospitalized with severe influenza. Preinfusion (d0; open symbols) and postinfusion (d1; closed symbols) median HAI titers (with interquartile range) against the B/Phuket/3073/2013 virus (**A**) and the A/California/07/09(H1N1) virus (**B**) are shown for the placebo- or Flu-IVIG–infused B/Yamagata-infected (Flu-IVIG *n* = 33 and placebo *n* = 29) and A/H1N1-infected (Flu-IVIG *n* = 24 and placebo *n* = 26) patients, respectively. Analysis of covariance, with the preinfusion or d0 level as a covariate, was used to compare differences between the placebo and Flu-IVIG treatment groups on d1 after infusion. The *P* value represents the difference between treatment groups for log_2_(d1 postinfusion titer), controlling for preinfusion (d0) titer. The dashed line represents an HAI titer of 40, which is considered an important protective threshold for influenza and is generally defined as 50% protective against influenza virus infection. ***P < 0.001.

HAI antibodies are a known correlate of protection against acquiring influenza virus infection, but their therapeutic potential is poorly understood. Treatment with Flu-IVIG led to an increase in HAI titers against the B/Phuket/3073/2013 virus (B/Phuket virus) in B/Yamagata-infected patients on d1 after infusion (median = 160; *P* < 0.001) compared with placebo (median = 40; Figure 3A). A significant rise in HAI titer against the A/California/07/2009 H1N1 virus (A/Cali09 virus) was also detected in A/H1N1-infected participants following Flu-IVIG infusion (median = 80; *P* < 0.001) relative to placebo (median = 10; Figure 3B). The treatment group difference between placebo and Flu-IVIG on d1 after infusion was adjusted for d0 baseline HAI titer. These results are in agreement with prior studies (8, 27), confirming that Flu-IVIG infusion causes a significant rise in HAI titer. Infusion with Flu-IVIG also led to an increase in neutralizing antibody titers (median = 160; *P* < 0.001) against the B/Phuket virus by microneutralization assay (MNA) and an increase in IgG against the B/Phuket HA stem (median fluorescence intensity [MFI] = 1010; *P* < 0.001) relative to placebo (median MNA titer = 20 and HA stem IgG MFI = 507; Supplemental Figure 1; supplemental material available online with this article; https://doi.org/10.1172/jci.insight.167464DS1).

### Flu-IVIG infusion increases Fc-functional antibodies in patients with influenza A and B.

**Figure 4:**
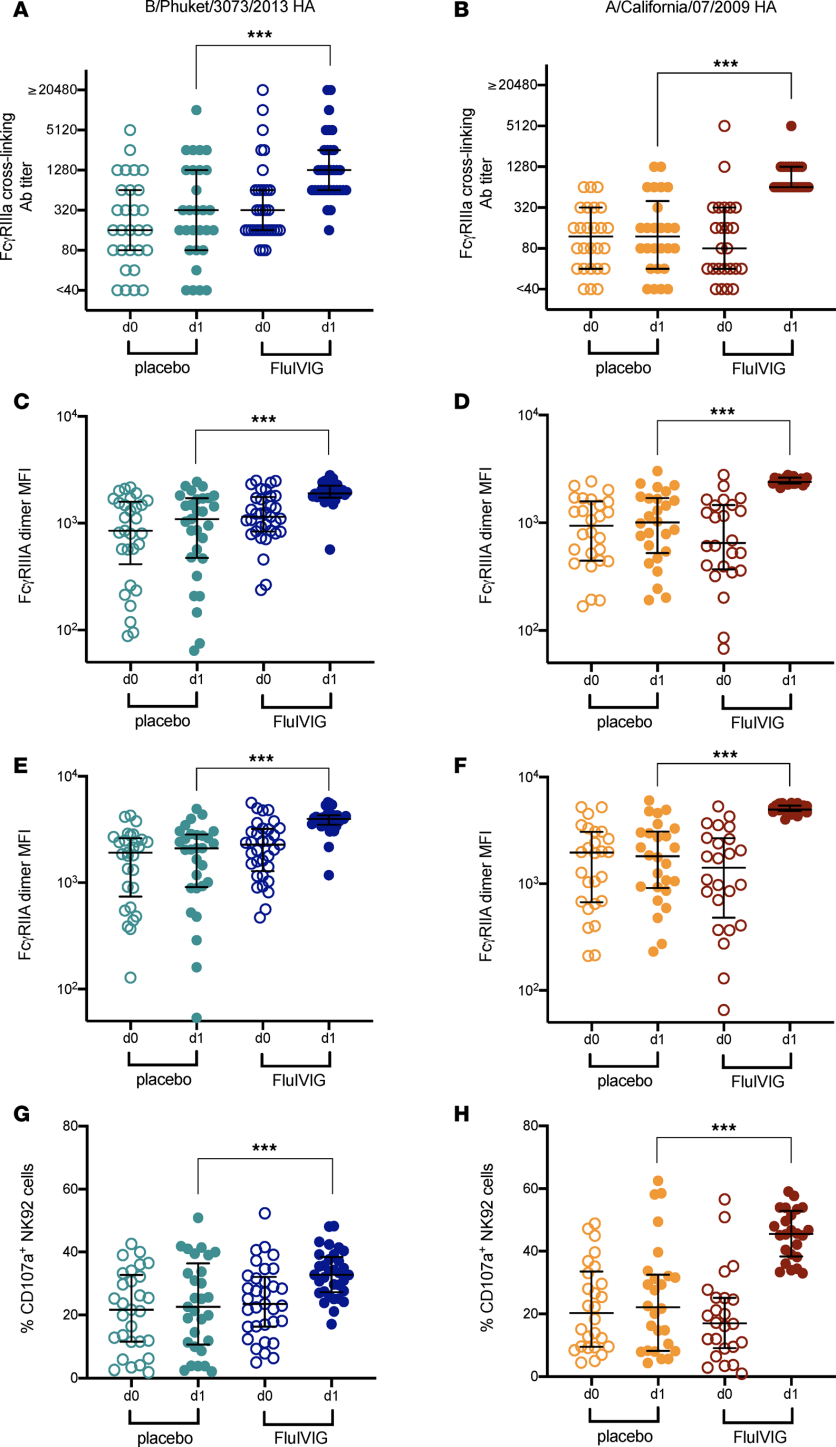
FcγR-binding antibody and antibody-dependent NK cell activation 1 day after infusion with influenza-specific hyperimmune immunoglobulin (Flu-IVIG) in patients hospitalized with severe influenza. The FcγRIIIa dimer ELISA (**A** and **B**), the FcγRIIIa and FcγRIIa dimer bead-based multiplex (**C**–**F**) and the plate-bound NK-92 cell activation assay (**G** and **H**) were used to analyze serum Fc functional antibody levels. Preinfusion (d0; open symbols) and postinfusion (d1; closed symbols) median (with interquartile range) FcγR dimer binding and NK cell activation (CD107a^+^) against the B/Phuket/3073/2013 HA (left figure panels) and the A/California/07/09(H1N1) HA (right figure panels) were measured for the placebo- or Flu-IVIG–treated B/Yamagata-infected (Flu-IVIG *n* = 33 and placebo *n* = 29) and A/H1N1-infected (Flu-IVIG *n* = 24 and placebo *n* = 26) patients, respectively. Analysis of covariance, with the preinfusion or d0 level as a covariate, was used to compare differences between the placebo and Flu-IVIG treatment groups on d1 after infusion. The *P* value represents the difference between treatment groups for log_2_(d1 postinfusion titer, MFI, or NK cell activation), controlling for preinfusion (d0) level. ***P < 0.001.

Passively infused antibodies with Fc effector functions can protect mice from lethal IAV and IBV infections (25, 28, 42), and we have shown that antibody engagement of recombinant human FcγRIIIa dimers correlates with antibody-dependent NK cell activation and ADCC activity in vitro (43, 44). We next investigated whether Flu-IVIG treatment increased FcγRIIIa- and FcγRIIa-binding antibodies as well as antibody-dependent NK cell activation. Infusion with Flu-IVIG boosted HA-specific FcγRIIIa cross-linking antibody titers (~4-fold) in B/Yamagata-infected patients (median = 1280; *P* < 0.001), compared with placebo titers (median = 320; Figure 4A). Similar results were obtained for A/H1N1-infected participants, with Flu-IVIG treatment resulting in an approximately 5-fold rise in HA-specific FcγRIIIa-binding antibodies (Flu-IVIG median d1 = 640 vs. placebo median d1 = 120; *P* < 0.001; Figure 4B). The treatment group difference between placebo and Flu-IVIG on d1 after infusion was adjusted for baseline d0 FcγRIIIa-binding antibody titer. Sera samples from B/Yamagata- and A/H1N1-infected patients demonstrated strong positive correlations between HA-specific FcγRIIIa dimer binding and HAI titers (Supplemental Figure 2), which supports our previous work (43, 45).

To confirm the above results, a bead-based multiplex assay was also used to assess both FcγRIIIa and FcγRIIa dimer–binding antibodies. In the B/Yamagata-infected patients, HA-specific FcγRIIIa- and FcγRIIa-binding antibodies were increased following infusion with Flu-IVIG (FcγRIIIa MFI = 1894 and FcγRIIa MFI = 3968) compared with placebo infusion (FcγRIIIa MFI = 1093 and FcγRIIa MFI = 2102, *P* < 0.001; Figure 4, C and D). A significant rise in HA-specific FcγRIIIa and FcγRIIa dimer–binding antibody was also detected in Flu-IVIG–treated A/H1N1-infected patients (FcγRIIIa MFI = 2398 and FcγRIIa MFI = 4961) relative to the placebo group (FcγRIIIa MFI = 1008 and FcγRIIa MFI = 1811, *P* < 0.001; Figure 4, E and F). As described above, treatment group differences between placebo and Flu-IVIG on d1 after infusion were adjusted for MFI of FcγR-binding antibody at baseline (d0).

To show that increased FcγR dimer binding leads to greater functional immune cell activation, an antibody-dependent NK cell activation assay was performed with INSIGHT 006 patient sera samples. We found that HA-specific NK cell activation was significantly increased in B/Yamagata- and A/H1N1-infected participants who received Flu-IVIG treatment (Figure 4, G and H). Strong positive correlations (*r* = 0.81–0.9, *P* < 0.001) were observed between HA-specific NK cell activation and FcγRIIIa dimer binding (ELISA and multiplex assay) for both B/Yamagata- and A/H1N1-infected patients (Supplemental Figure 3).

There is increasing interest in anti-NA antibodies, as they are also capable of blocking virion egress and mediating Fc effector functions. A rise in NA-specific FcγRIIIa and FcγRIIa binding was detected following treatment with Flu-IVIG in B/Yamagata-infected patients (Supplemental Figure 4), which closely mirrored data for influenza HA.

### IgG subclass analysis in Flu-IVIG–treated IAV- and IAB-infected patients.

Different IgG subclasses selectively engage human FcγRs, with IgG1 and IgG3 having the greatest affinity for activating human FcγRs, like FcγRIIIa and FcγRIIa (38, 39). To further characterize the impact of Flu-IVIG treatment on humoral immunity in patients with severe influenza A or B, we examined changes in influenza-specific IgG subclasses. In B/Yamagata-infected patients, higher levels of IgG1, IgG2, and IgG3 against the HA of the reference B/Phuket virus were detected following Flu-IVIG infusion (IgG1 MFI = 18,530, IgG2 MFI = 321, and IgG3 MFI = 325) compared with placebo controls (IgG1 MFI = 8024, IgG2 MFI = 75, and IgG3 MFI = 137; Supplemental Figure 5, A, C, and E). In A/H1N1-infected patients, higher levels of HA-specific IgG1, IgG2, and IgG3 were also detected following Flu-IVIG infusion (IgG1 MFI = 23,412, IgG2 MFI = 76, and IgG3 MFI = 434) relative to placebo controls (IgG1 MFI = 15,125, IgG2 MFI = 42, and IgG3 MFI = 44; Supplemental Figure 5, B, D, and F). There was no difference in HA-specific IgG4 between the placebo- and Flu-IVIG–treated groups and no detectable increase after Flu-IVIG treatment (Supplemental Figure 5, G and H). Due to its low abundance in sera, IgG4 in patient samples was often below the limit of detection of the multiplex assay following subtraction of blank and background wells (B/Yamagata: 11/124 undetectable and A/H1N1: 14/100 undetectable). Anti-NA IgG1, IgG2, and IgG3 were also significantly increased after infusion with Flu-IVIG in B/Yamagata- and A/H1N1-infected participants relative to placebo and preinfusion levels (Supplemental Figures 6 and 7). NA-specific IgG3 and IgG4 levels were very low and frequently undetectable in the multiplex assay (IgG3: 82/224 undetectable and IgG4: 174/224 undetectable; Supplemental Figures 6 and 7).

### Association between anti-HA antibody titer and outcome in IAV- and IBV-infected patients.

To determine whether high anti-HA antibody titers were associated with improved d5 clinical outcomes following Flu-IVIG or placebo infusion, the B/Yamagata- and A/H1N1-infected patients were divided into low- and high-antibody-titer groups and then analyzed using univariate or multivariate proportional odds regression models. The anti-HA antibody titers were generated by HAI assays and HA-specific FcγRIIIa dimer binding ELISAs. For the analysis, the Flu-IVIG and placebo groups were pooled to perform a whole-cohort assessment of whether high- or low-antibody-titer groups are associated with better influenza outcome regardless of the source of antibody, either artificially infused (for those who received Flu-IVIG) or naturally mounted due to infection (for those who received placebo). Summary ORs greater than 1 indicate that the high-antibody-titer group has better odds of being in a more favorable clinical outcome category, whereas ORs less than 1 favor the low-antibody-titer group.

**Figure 5:**
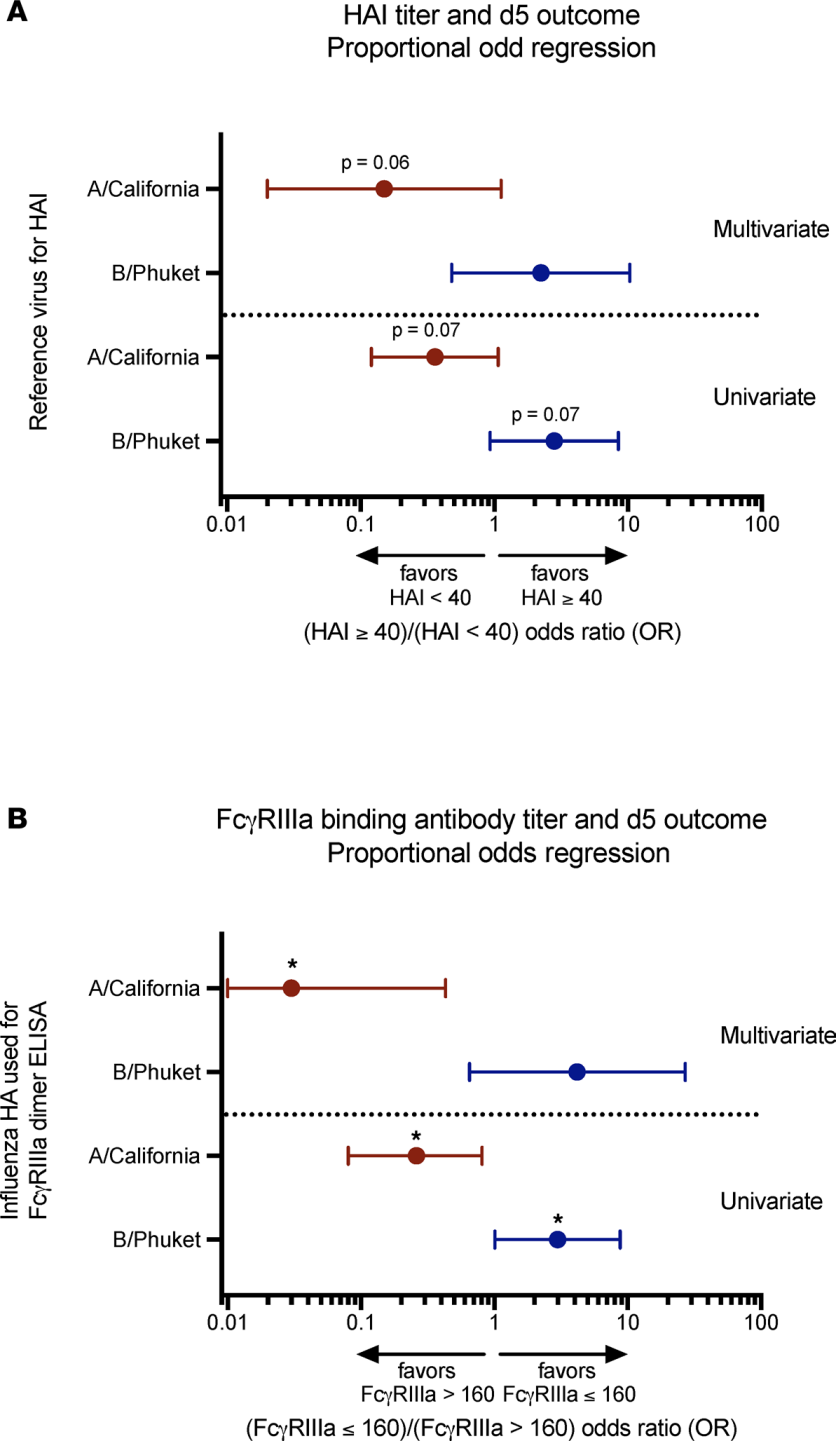
Association between clinical outcome and HAI or FcγRIIIa-binding antibody titer in B/Yamagata- and A/H1N1-infected patients. (**A**) Based on postinfusion (d1) serum titers, patients with severe B/Yamagata (*n* = 62; blue) and A/H1N1 (*n* = 50; red) influenza were divided into high (≥40) and low (<40) HAI titer groups irrespective of randomization to Flu-IVIG or placebo. (**B**) Patients with severe B/Yamagata (*n* = 62; blue) and A/H1N1 (*n* = 50; red) influenza were also divided into high (>160) and low (≤160) FcγRIIIa-binding antibody titer groups regardless of treatment group. The association between HAI or FcγRIIIa-binding antibody titer and d5 postinfusion ordinal outcomes was investigated using univariate and multivariate proportional odds regression models, with the multivariate model adjusting for baseline antibody titer, treatment group (Flu-IVIG/placebo), and risk score on d7. Odds ratios (ORs) are shown with 95% confidence intervals, with ORs greater than 1 indicating that the high-antibody-titer group has better odds of being in a more favorable clinical outcome category and ORs less than 1 favoring the low-antibody-binding group. *P <0.05.

An HAI titer of 40 is typically defined as 50% protective against influenza virus infection (46, 47). In patients with severe B/Yamagata infection, there was a trend toward participants with postinfusion HAI titers equal to or greater than 40 having better odds of a favorable d5 ordinal outcome compared with those with HAI titers less than 40 in a univariate proportional odds regression model (OR = 2.8, 95% CI = 0.9–8.5; *P* = 0.07), but this trend was not observed in a multivariate model (OR = 2.2, 95% CI = 0.5–10.3; *P* = 0.30; Figure 5A). In participants with severe A/H1N1 influenza, there was a trend toward patients with postinfusion HAI titers of 40 or greater having poorer odds of a favorable d5 ordinal outcome in both the univariate (OR = 0.36, 95% CI = 0.1–1.1; *P* = 0.07) and the multivariate (OR = 0.2, 95% CI = 0.02–1.1; *P* = 0.06) proportional odds regression models, but none of the observed differences were statistically significant (Figure 5A).

High preexisting ADCC antibody titers (>160) have previously been reported to reduce influenza disease severity in an experimental human influenza challenge (35). In this study, IBV-infected patients with high postinfusion HA-specific FcγRIIIa cross-linking antibody titers (>160) had better odds of a favorable d5 ordinal outcome compared with patients with lower titers in a univariate proportional odds regression model (OR = 2.98, 95% CI = 1.0–8.8; *P* = 0.048), but significance was lost in the multivariate model (OR = 4.2. 95% CI = 0.7–27.1; *P* = 0.13; Figure 5B). Poorer odds of a favorable clinical outcome were observed in A/H1N1-infected patients with high FcγRIIIa cross-linking antibody titers compared with participants with lower titers in both univariate (OR = 0.3, 95% CI = 0.1–0.8; *P* = 0.02) and multivariate (OR = 0.03, 95% CI = 0.01–0.4; *P* = 0.01) proportional regression models (Figure 5B).

### Association between anti-influenza antibody features and clinical outcomes in patients with severe influenza A and B.

To investigate whether antibody characteristics (including IgG and IgA subclasses, FcγR binding, HAI, or NK cell activation) are associated with better outcomes, univariate and multivariate proportional odds regression models were performed. Antibody isotype and subclass composition as well as FcγR binding were measured using bead-based multiplex assays. The B/Yamagata- and A/H1N1-infected patients were analyzed separately, with the placebo- and Flu-IVIG–treated participants pooled to evaluate associations between the antibody parameters tested and clinical outcome irrespective of antibody source. Summary ORs greater than 1 suggest that patients with higher antibody levels have improved odds of being in a better clinical outcome category, whereas ORs less than 1 indicate that patients with lower antibody levels have improved odds of being in a better outcome category.

**Figure 6:**
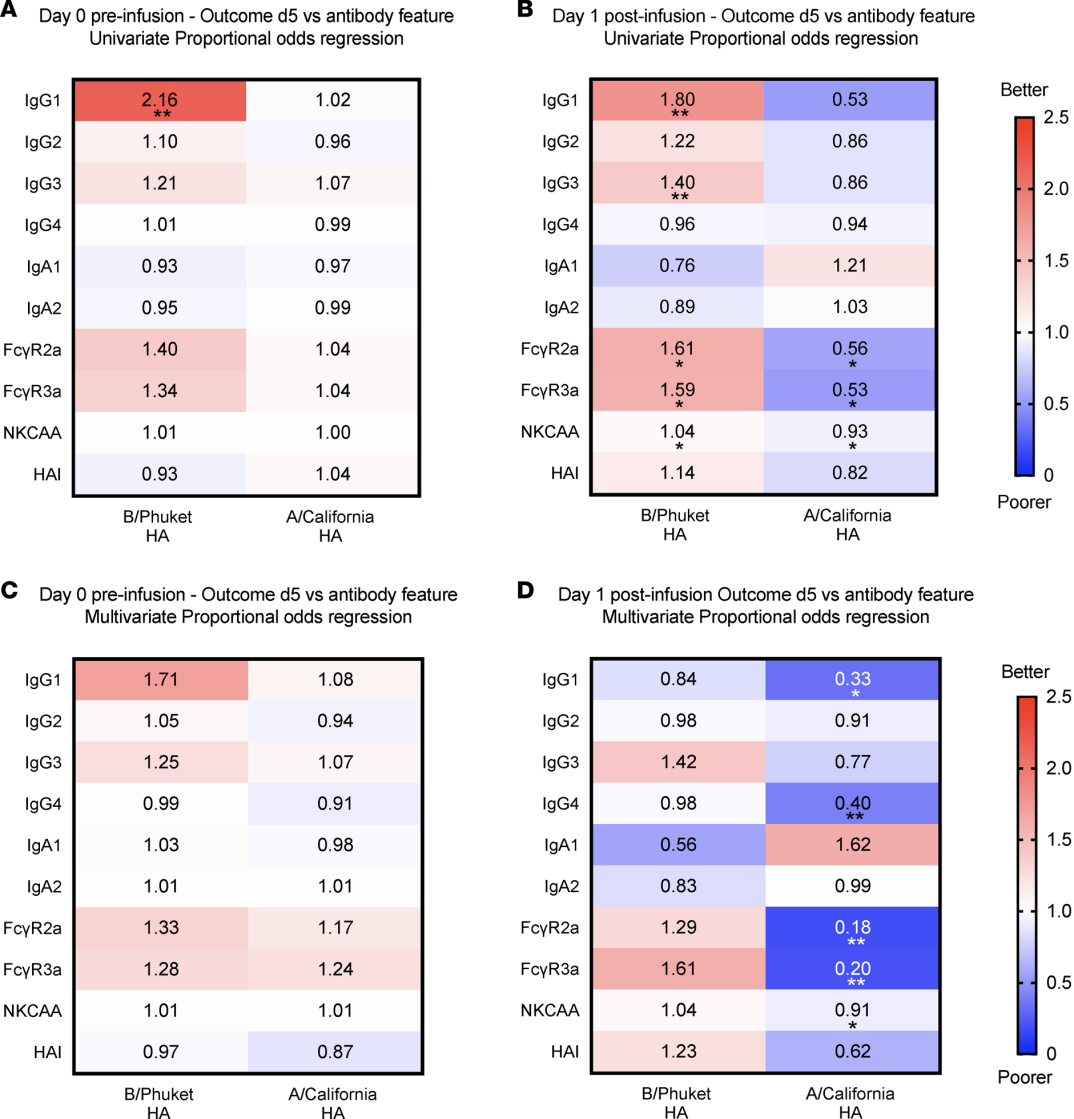
Associations between influenza antibody features and clinical outcomes on day 5 after infusion in patients hospitalized with severe B/Yamagata and A/H1N1 influenza. The association between antibody features and day 5 (d5) postinfusion ordinal outcomes were investigated using univariate and multivariate proportional odds regression models, with the multivariate model adjusting for baseline antibody level, treatment group (Flu-IVIG/placebo), and risk score on d7. Heatmaps show summary odds ratios (ORs) for patients hospitalized with B/Yamagata (*n* = 62; left side of heatmap) and A/H1N1 (*n* = 50; right side of heatmap) influenza at preinfusion (**A** and **C**) and d1 postinfusion (**B** and **D**) time points generated using univariate (**A** and **B**) and multivariate (**C** and **D**) proportional odds regression models. ORs greater than 1 indicate that patients with higher antibody levels have improved odds of being in a better outcome category on d5 after infusion and ORs less than 1 indicating that patients with lower antibody levels have improved odds of being in a better outcome category. *P <0.05, **P <0.01.

At the baseline preinfusion time point (d0), HA-specific IgG1 was associated with a more favorable outcome in B/Yamagata-infected patients (Figure 6, A and C; left side of heatmap). Following Flu-IVIG or placebo infusion (d1 after infusion), HA-specific IgG1, IgG3, FcγRIIa-binding antibody, FcγRIIIa-binding antibody, and NK cell activation were associated with improved odds of a favorable d5 ordinal outcome in patients with severe influenza B using a univariate proportional odds regression model (Figure 6B; left side of heatmap). In a multivariate regression model, none of the antibody features tested were significantly associated with more favorable d5 ordinal outcomes in patients with severe B/Yamagata influenza, although HA-specific IgG3 (OR = 1.42), FcγRIIa-binding antibody (OR = 1.29), and FcγRIIIa-binding antibody (OR = 1.61) still had OR values greater than 1 in the adjusted model (Figure 6D; left side of heatmap). In the participants with influenza B, a trend suggesting a potential association between postinfusion HA-specific IgG1 and poorer odds of a favorable d3 clinical outcome was shown only in the multivariate proportional odds regression model (OR = 0.53, *P* = 0.06; Supplemental Figure 8), but this trend was not observed in the univariate model nor at later d5 and d7 time points (Figure 6 and Supplemental Figure 9).

In the A/H1N1-infected patients, none of the antibody features tested were associated with d5 clinical outcomes at the preinfusion time point (Figure 6, A and C; right side of heatmap). Interestingly, postinfusion HA-specific FcγRIIa-binding antibody, FcγRIIIa-binding antibody, and NK cell activation were associated with worse odds of a favorable d5 clinical outcome in patients with severe A/H1N1 influenza using both univariate and multivariate proportional odds regression models (Figure 6, B and D; right side of heatmap). Proportional odds regression models were also performed for ordinal outcomes on d3 (Supplemental Figure 8), d7 (Supplemental Figure 9), and for B/Phuket NA–specific antibodies (Supplemental Figure 10), with similar trends observed as for the d5 outcomes overall.

### Flu-IVIG treatment reduces odds of a favorable clinical outcome in A/H1N1-infected patients with low baseline FcγRIIIa-binding antibody titers.

Antibody-based therapies like Flu-IVIG may be of greatest benefit in patients who do not mount a robust humoral response to infection (48). Subgroup analyses were, therefore, performed to determine whether Flu-IVIG treatment improved the odds of a more favorable clinical outcome in patients with below- or above-median baseline (d0 preinfusion) HA-specific FcγRIIIa-binding antibody titers. Summary ORs greater than 1 indicate that patients infused with Flu-IVIG have better odds of being in a more favorable clinical outcome category, whereas ORs less than 1 favor placebo infusion.

**Figure 7:**
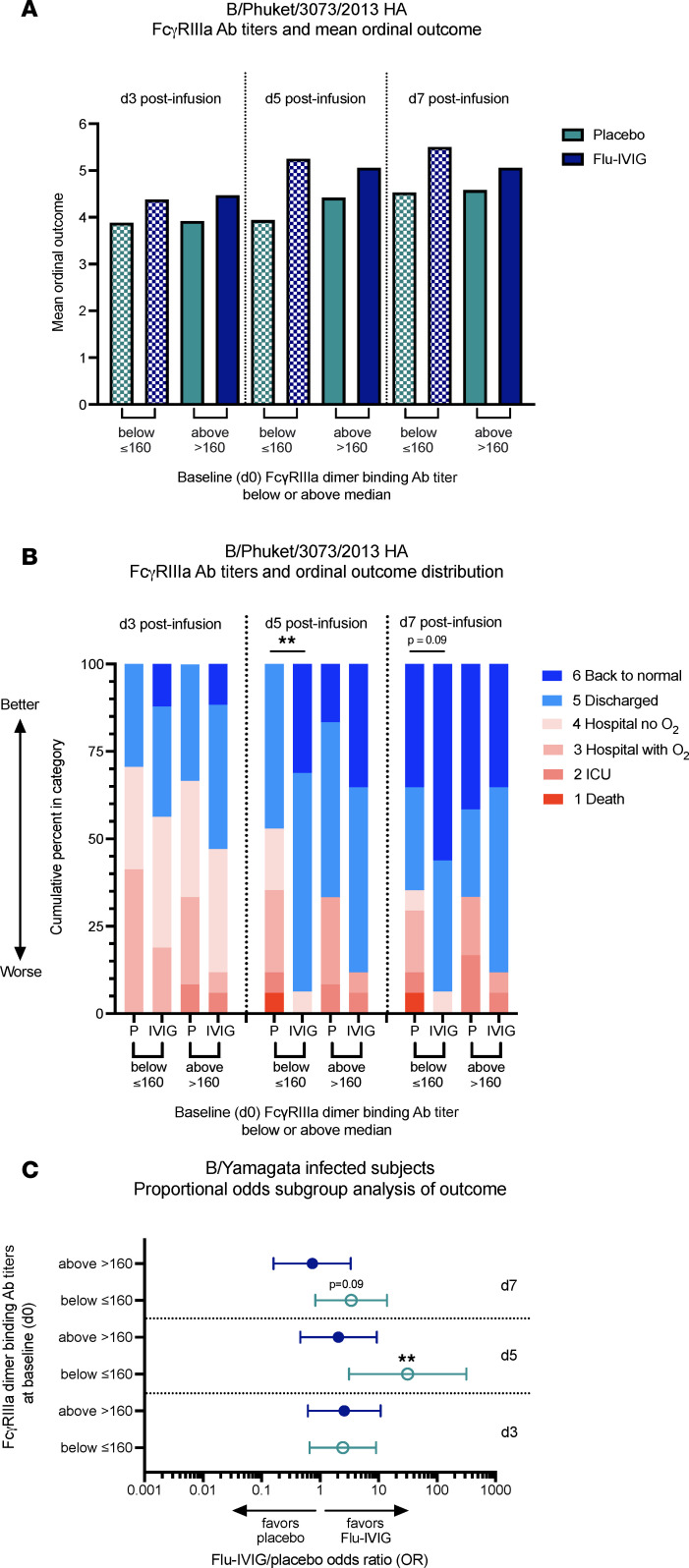
Treatment effect of Flu-IVIG in B/Yamagata-infected patients with below- and above-median baseline FcγRIIIa-binding antibody titers. Placebo- (*n* = 29) and Flu-IVIG–treated (*n* = 33) B/Yamagata-infected patients were grouped by below-median (≤160) or above-median (>160) baseline (or preinfusion) FcγRIIIa-binding antibody titer. Mean ordinal outcomes (**A**) and ordinal outcome distributions (**B**) on day 3 (d3), d5, and d7 after infusion are shown for the Flu-IVIG and placebo groups with below- (≤160) or above-median (>160) baseline FcγRIIIa-binding antibody titers. (**C**) The Flu-IVIG/placebo odds ratios (ORs) with 95% confidence intervals are shown for the below- and above-median FcγRIIIa-binding antibody titer subgroups. A proportional odds regression model with adjustment for the patient’s baseline clinical status was used to compare the Flu-IVIG– and placebo-treated subgroups. ORs greater than 1 indicate that patients infused with Flu-IVIG have better odds of being in a more favorable clinical outcome category, whereas ORs less than one favor placebo infusion. **P <0.01.

Overall, Flu-IVIG infusion showed more favorable mean ordinal outcomes and ordinal outcome distributions, relative to placebo, in B/Yamagata-infected patients with below-median (≤160) and above-median (>160) baseline FcγRIIIa-binding antibody titers (Figure 7, A and B). Treatment with Flu-IVIG improved the odds of a more favorable clinical outcome on d3 (OR = 2.5, 95% CI = 0.7–9.0), d5 (OR = 31.5, 95% CI = 3.1–316.2), and d7 (OR = 3.4, 95% CI = 0.8–13.9) after infusion in B/Yamagata-infected patients with below-median (≤160) baseline FcγRIIIa-binding antibody titers, and statistical significance was reached on d5 after infusion (*P* = 0.003; Figure 7C). There was no treatment effect of Flu-IVIG in B/Yamagata-infected patients with above-median (>160) baseline FcγRIIIa-binding antibody titers (Figure 7C). The treatment effect of Flu-IVIG versus placebo was not significantly different between the below-median (≤160) and the above-median (>160) subgroups; however, a trend was observed on d5 after infusion (interaction *P* = 0.10). These findings suggest that IBV-infected participants with lower baseline Fc-functional antibody titers may be receiving some additional clinical benefit from Flu-IVIG treatment.

**Figure 8:**
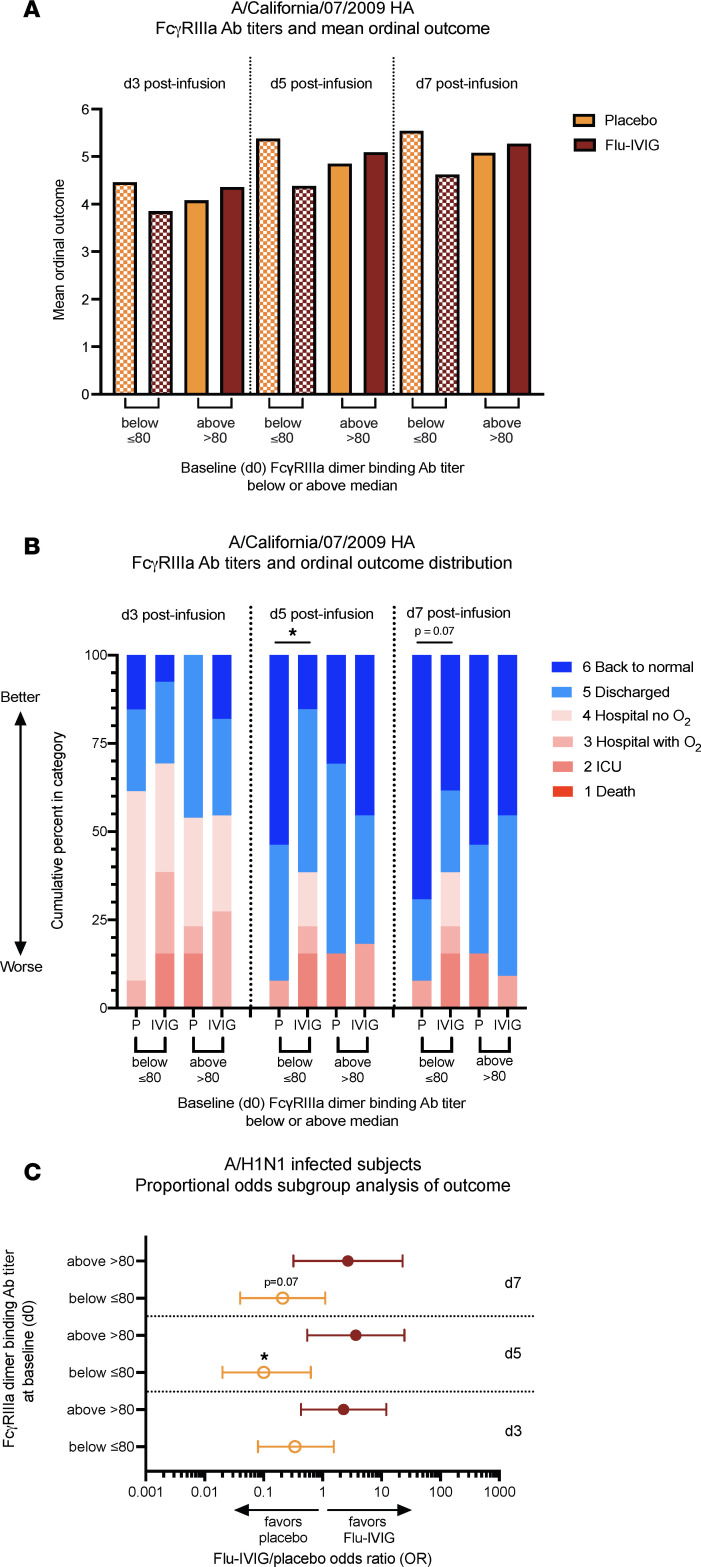
Treatment effect of Flu-IVIG in A/H1N1-infected patients with below- and above-median baseline FcγRIIIa-binding antibody titers. Placebo- (*n* = 26) and Flu-IVIG–treated (*n* = 24) A/H1N1-infected patients were grouped into below-median (≤80) or above-median (>80) baseline (or preinfusion) FcγRIIIa-binding antibody titer. Mean ordinal outcomes (**A**) and ordinal outcome distributions (**B**) on day 3 (d3), d5, and d7 after infusion are shown for the Flu-IVIG– and placebo-infused patients with below-median (≤80) or above-median (>80) baseline FcγRIIIa-binding antibody titers. (**C**) The Flu-IVIG/placebo odds ratios (ORs) with 95% confidence intervals are shown for the below- and above-median FcγRIIIa-binding antibody titer subgroups. A proportional odds model with adjustment for the patient’s baseline clinical status was used to compare the Flu-IVIG– and placebo-treated subgroups. ORs greater than 1 indicate that patients infused with Flu-IVIG have better odds of being in a more favorable clinical outcome category, whereas ORs less than 1 favor placebo infusion. *P <0.05.

Surprisingly, Flu-IVIG infusion showed less favorable mean ordinal outcomes and ordinal outcome distributions compared with placebo infusion in A/H1N1-infected patients with below-median (≤80) baseline FcγRIIIa-binding antibody titers (Figure 8, A and B). Mean ordinal outcomes and ordinal outcome distributions were similar in placebo and Flu-IVIG groups for A/H1N1-infected patients with above-median (>80) baseline FcγRIIIa-binding antibody titers (Figure 8, A and B). Treatment with Flu-IVIG reduced the odds of a more favorable outcome in A/H1N1-infected patients with below-median (≤80) baseline FcγRIIIa-binding antibody titers on d3 (OR = 0.3, 95% CI = 0.1–1.6), d5 (OR = 0.1, 95% CI = 0.02–0.6), and d7 (OR = 0.2, 95% CI = 0.04–1.1) after infusion, and the odds of a more favorable outcome were significantly worse than placebo on d5 (*P* = 0.01; Figure 8C). There was no treatment effect of Flu-IVIG in A/H1N1-infected patients with above-median (>80) baseline FcγRIIIa-binding antibody titers (Figure 8C). The treatment effect of Flu-IVIG versus placebo was significantly different between the below-median (≤80) and the above-median (>80) subgroups on d5 after infusion (interaction *P* = 0.004) and d7 after infusion (interaction *P* = 0.04). Thus, Flu-IVIG treatment was associated with less favorable outcomes in A/H1N1-infected participants who had lower levels of Fc-functional antibodies at baseline.

### Flu-IVIG treatment decreases serum concentration of IL-6 in patients with severe influenza.

Participant serum was tested for several proinflammatory cytokines, including IL-6, IL-1β, ΤΝF-α, and IFN-γ. For B/Yamagata- and A/H1N1-infected patients, serum cytokine levels were generally low or below the limit of detection for IL-1β, ΤΝF-α, and IFN-γ, precluding any further analyses of the treatment groups (data not shown). Patients with severe B/Yamagata influenza had slightly higher concentrations of serum IL-6 than patients with A/H1N1 influenza in both the placebo and Flu-IVIG groups at preinfusion (d0) and postinfusion (d1) time points. Serum concentrations of IL-6 were significantly lower for Flu-IVIG–infused patients, relative to placebo, for both A/H1N1- and B/Yamagata-infected patients on d1 after infusion (*P* < 0.001; Supplemental Figure 11).

## Discussion

The development of antibody-based therapies and prophylactics for severe human influenza has been a long-standing but elusive aim of influenza researchers worldwide. No antibody-based treatments are available for patients hospitalized with severe IAV or IBV infections (17), despite a large number of antibody-based therapies being tested in preclinical animal models and clinical trials (4–21). In recent years, broadly neutralizing mAbs targeting the HA stem have been a major focus of universal influenza therapy design. However, many of the promising anti–HA stem mAbs did not reduce symptoms or time to viral clearance in efficacy trials (4, 6, 11, 17). An alternative approach is to treat severe human influenza with polyclonal antibody mixtures, such as convalescent plasma and hyperimmune IVIG. Convalescent blood products appeared to reduce mortality following severe infections with both the A/H1N11918 and 2009 pandemic IAVs (1–3), although the relative lack of rigor of some prior trials has meant that these treatments have remained in the research arena.

A recent placebo-controlled phase III clinical trial (INSIGHT 006) revealed that infusion with Flu-IVIG was beneficial for patients hospitalized with severe influenza B (8). Surprisingly, Flu-IVIG did not provide any clinical benefit for patients hospitalized with influenza A, in spite of a robust rise in HAI titer following treatment (8). To explore a potential role for Fc-mediated antibody functions in improving outcomes during severe influenza B, serum antibodies from B/Yamagata- and A/H1N1-infected patients enrolled in the Flu-IVIG trial were analyzed. We first showed that Flu-IVIG treatment increased serum HAI titers in the A/H1N1- and B/Yamagata-infected patients relative to placebo-infused controls, which mirrored results from the larger INSIGHT 006 clinical trial (8). Following Flu-IVIG infusion, B/Yamagata-infected patients had a 2-fold higher median HAI titer (median HAI titer = 160 against the B/Phuket virus) than A/H1N1 patients (median HAI titer = 80 against the A/Cali09 virus), which may play a role in the observed protection in influenza B. However, participants with influenza B also showed 2- to 4-fold higher median HAI titers before infusion with Flu-IVIG and in the placebo-treated group. The higher HAI titers in patients with severe influenza B may be due to limited antigenic drift and greater relative stability of IBVs compared with IAVs (49). Patients with severe influenza B who received Flu-IVIG had higher neutralizing antibody titers (by MNA) and higher levels of HA stem–specific IgG compared with placebo recipients, both of which may also contribute to the clinical benefit of Flu-IVIG treatment in patients with influenza B. While HAI titer is a known correlate of protection against acquiring influenza virus infection (46, 47), the therapeutic benefit of infusing HAI and neutralizing antibodies following influenza symptom onset is unclear. In this study, Flu-IVIG was administered up to 7 days after symptom onset (8, 50), once influenza virus infection was already established. The HAI antibodies in Flu-IVIG also have limited therapeutic applications, as they may not be well matched against antigenically drifted or emerging influenza viruses. Antibodies with Fc effector functions target more conserved HA epitopes than HAI antibodies and are important for clearance of influenza virus–infected cells. We have previously shown that Flu-IVIG preparations contain Fc-functional antibodies that can bind to a broad range of influenza virus strains and subtypes (27).

In the Flu-IVIG preparations, IBV antibodies were reported to have higher affinities than the IAV antibodies, resulting in slower dissociation rates of immune complexes (8). Since the functionality of immune complexes depends heavily on engagement of FcγRs expressed by immune cells, we next measured FcγR-binding antibodies in participant sera. We found that Flu-IVIG treatment significantly increased HA-specific FcγRIIIa- and FcγRIIa-binding antibodies and resulted in greater NK cell activation compared with placebo in patients hospitalized with both B/Yamagata and A/H1N1 influenza. Patients with B/Yamagata influenza virus had a 2-fold higher median HA-specific FcγRIIIa-binding antibody titer (median titer = 1280 against the B/Phuket HA) than A/H1N1 patients (median titer = 640 against the A/Cali09 HA) following Flu-IVIG treatment, and this may contribute to Flu-IVIG protection in patients with influenza B. Similar to the HAI results, influenza B participants also showed 2- to 4-fold greater FcγRIIIa-binding antibody titers before infusion with Flu-IVIG and after placebo infusion. As noted above, the elevated FcγR-binding antibody levels observed for patients with influenza B may be the result of reduced antigenic drift and higher stability of IBVs relative to IAVs (49). Fc-mediated effector functions are required for broadly reactive anti-HA mAbs to confer protection from lethal IAV and IBV infections in a murine passive infusion model (25, 28, 42). Furthermore, ferrets infused with IVIG were protected from lethal challenge with an H5N1 influenza virus in the absence of detectable HAI antibodies (51). A small human influenza challenge study also showed that high levels of ADCC-mediating antibodies were associated with decreased viral shedding and reduced disease severity (35). Together, these studies suggest that the rise in anti-HA and anti-NA Fc-functional antibodies following Flu-IVIG treatment may contribute to improving clinical outcomes in patients with severe influenza B.

The majority of influenza challenge studies demonstrating a protective role for Fc-mediated antibody functions have been performed using IAVs (25, 28). However, higher levels of Fc-functional antibodies after Flu-IVIG treatment did not show any clinical benefit for patients with influenza A. While Fc-mediated effector functions are known to be protective in murine models of influenza, Fc-functional antibodies can also play a role in immunopathology and inflammation during viral infections (52). The delicate balance between protective and pathological roles of Fc-functional antibodies may be impacted by the type, subtype, or strain of influenza virus infection, clinical presentation, and host immunological factors, including preexisting immunity. Humans, unlike animal models, have a complex history of influenza virus exposure resulting in a broad range of preexisting Fc-functional antibodies and memory responses (33–35), which may impact clinical outcomes irrespective of Flu-IVIG treatment. A limitation of the INSIGHT 006 trial is that the majority of IBV-infected participants were recruited in the last 1.5 years of the trial; therefore, some immunological differences between the IAV- and IBV-infected populations may exist, and this could impact responsiveness to Flu-IVIG treatment. Further investigation is necessary to dissect the mechanisms underlying the differential outcomes of Flu-IVIG therapy in patients hospitalized with IAV or IBV.

Antibody characteristics, including IgG1, IgG3, FcγRIIIa binding, FcγRIIa binding, and NK cell activation, were associated with better clinical outcomes in participants with severe influenza B in a univariate proportional odds regression model, but the significance of these associations was reduced in the multivariate model and needs to be confirmed with larger trials. These preliminary findings suggest that Fc-functional antibodies may play a more protective role in IBV infection by assisting to reduce disease severity through mechanisms such as antibody-dependent phagocytosis (ADP) and ADCC. No significant association between HAI titer and clinical outcome were observed for patients infected with B/Yamagata or A/H1N1, so it is unclear whether neutralization of free virions is playing any protective role. Cross-lineage anti-HA mAbs protect mice from lethal IBV infection either by targeting the receptor binding domain or by non-neutralizing Fc-mediated functions (42). Polyfunctional humoral immunity and elevated levels of FcγR-engaging antibody may also be associated with better clinical outcomes in humans hospitalized with severe influenza B, but a larger clinical trial would be required to fully investigate this.

Interestingly, several antibody features were associated with worse outcomes in patients hospitalized with influenza A. Higher levels of HA-specific FcγR dimer–binding antibody and NK cell activation were associated with poorer clinical outcomes in patients with severe A/H1N1 influenza, indicating that the immunopathological or inflammatory effects of Fc-functional antibodies may outweigh their protective effects in this instance. In experimental IAV challenge studies, higher levels of HA-specific ADCC antibodies are often detected in humans with more severe influenza symptoms (35, 53), but whether this is simply due to greater viral replication and antigen availability is not clear. FcγR cross-linking antibodies can clear infected cells and help control viral replication, but these antibodies may also contribute to inflammation and immunopathology at the site of infection. High levels of non-neutralizing antibodies and pathogenic immune complexes (formed with these non-neutralizing antibodies) were identified in patients with severe influenza and fatal infections with the A/H1N1 2009 pandemic IAV (54, 55). Mice immunized with an ADCC epitope (E1), found in the HA head domain, had increased alveolar damage and mortality following infection with the A/H1N1 2009 pandemic IAV compared with PBS-immunized mice (52). Vaccination with the E1 epitope significantly increased proinflammatory cytokines and perforin in the murine lungs 5 days after infection, suggesting that ADCC may be driving excessive inflammation and immune cell infiltration in the lungs (52). While E1-vaccinated mice did show a modest decrease in lung viral load, any protective effect was outweighed by inflammatory lung damage (52). Serum concentrations of proinflammatory cytokines, including IL-1β, ΤΝF-α, and IFN-γ, were low or below the limit of detection in most study participants irrespective of treatment group or influenza type. There was a reduction in serum IL-6 in the Flu-IVIG–treated group, compared with the placebo group, for patients with severe influenza A and B on d1 after infusion. This indicates Flu-IVIG treatment may reduce systemic inflammation shortly after infusion, but serum IL-6 does not necessarily reflect local lung inflammation at later postinfusion time points (d5 and d7). Further studies with more direct measures of lung inflammation are needed. The observed associations between high levels of Fc-functional antibody and poorer outcomes in patients with severe A/H1N1 influenza suggest the hypothesis that antibody-induced inflammation may drive immunopathology in the IAV-infected human lung.

Antibody-based therapies often provide the greatest benefit for patients who do not mount a rapid or effective humoral response to infection. As such, we performed subgroup analyses to determine the impact of Flu-IVIG infusion on patients with below or above median FcγRIIIa-binding titers. In patients hospitalized with B/Yamagata influenza, the Flu-IVIG–treated participants with lower (or below median) baseline Fc-functional antibody titers had improved clinical outcomes relative to placebo on d5 after infusion, with an ongoing trend toward improved outcomes on d7 after infusion. These results suggest that IBV-infected participants with low baseline levels of Fc-functional antibody may be receiving greater clinical benefit following Flu-IVIG therapy, possibly due to the infusion of ADCC- and ADP-mediating antibodies that can remove virus-infected cells and free virions. Additional mechanistic studies in animal models are required to pinpoint the immune cells and effector functions involved in the protective effect of Flu-IVIG against IBV. Furthermore, future clinical trials specifically targeted toward severe influenza B are needed to validate the observed clinical benefit of Flu-IVIG therapy in this subgroup.

The effect of Flu-IVIG therapy was different in patients hospitalized with severe influenza A. For the A/H1N1-infected group, Flu-IVIG–treated patients with low (or below median) baseline titers of FcγRIIIa-binding antibody had significantly worse outcomes on d5 after infusion compared with placebo, with this trend continuing on d7 after infusion. This is consistent with the association between poorer clinical outcomes and higher levels of HA-specific Fc-functional antibody. Taken together, these findings suggest the hypothesis that high levels of FcγR-binding and NK cell–activating antibody may be worsening clinical outcomes by driving excessive inflammation in the lungs of IAV-infected participants or by some other unknown mechanism. Few studies on Fc-mediated antibody functions have examined their ability to enhance immunopathology (52, 54–56). In vivo knockout and immune cell depletion studies in animal models are needed to dissect the mechanisms that underpin potential Fc-mediated immunopathology during severe IAV infection.

Overall, HAI and Fc-functional antibodies were both increased following Flu-IVIG treatment in the A/H1N1- and B/Yamagata-infected participants. Antibody characteristics such as IgG3 and FcγR engagement showed some association with improved odds of a more favorable outcome in patients with severe influenza B. Unexpectedly, FcγR-binding antibody and antibody-dependent NK cell activation were associated with poorer odds of a favorable clinical outcome in the A/H1N1-infected participants after infusion. Treatment with Flu-IVIG improved the odds of a more favorable clinical outcome in B/Yamagata-infected patients with lower baseline levels of Fc-functional antibody. In contrast, treatment with Flu-IVIG worsened the odds of a more favorable clinical outcome in A/H1N1-infected patients with lower levels of baseline Fc-functional antibody. Our comprehensive examination of serum antibodies from Flu-IVIG– and placebo-infused humans with severe influenza has provided invaluable insight into the mechanisms and antibody characteristics that underpin effective humoral immunity against influenza virus. This knowledge will help to inform the development of new and improved antibody-based therapies to reduce the health burden of severe human influenza.

## Methods

### Flu-IVIG.

The 5 Flu-IVIG batches given to patients were manufactured annually from 2013 to 2017 inclusive (for use from 2014 to 2018 in the INSIGHT 006 clinical trial) by Emergent BioSolutions under contract with the NIH (8). The Flu-IVIG lots were prepared using anti-influenza plasma collected from fractionated whole blood or by plasmapheresis from influenza-immune volunteers at designated collection sites in the United States and Canada. Influenza-immune volunteers and plasma units were selected on the basis of elevated HAI titers against contemporary vaccine strains (for further details see ref. 8).

### HA and NA proteins.

In the INSIGHT 006 clinical trial, the A/Cali09 virus and the B/Phuket virus were the major reference strains studied in the A/H1N1- and B/Yamagata-infected patients, respectively (8). These reference strains were recommended by the WHO for inclusion in the seasonal influenza vaccine from the 2013/14 to the 2016/17 influenza seasons for the A/Cali09 virus and from the 2015/16 to the 2017/18 influenza seasons for the B/Phuket virus, indicating that they were the predominant strains circulating in the human population during the bulk of the clinical trial period. Therefore, the majority of our study cohort, including both the A/H1N1 and B/Yamagata groups, were enrolled during influenza seasons where their reference virus (A/Cali09 or B/Phuket respectively) was recommended for inclusion in the seasonal vaccine. As such, we performed all assays with the A/Cali09 and B/Phuket reference viruses or HA proteins from these viruses to reflect, as closely as feasible, the antibody responses of the B/Yamagata- and A/H1N1-infected participants recruited. Recombinant HA proteins from the A/Cali09 virus and the B/Phuket virus, NA protein from the B/Phuket virus, and a negative control protein simian immunodeficiency virus gp120 (SIV gp120) were purchased from SinoBiological. A stabilized HA stem protein derived from the B/Phuket virus (42, 57) and a stabilized NA protein (SNAP) from the A/Cali09 virus (58) were designed as previously described and provided by Adam Wheatley (University of Melbourne, Parkville, Australia).

### HAI assay.

HAI assays for A/California/07/2009 H1N1 and B/Phuket/3073/2013 viruses were performed as previously described (59). The B/Phuket/3073/2013 virus was ether split to perform the HAI assay (60). The MNAs were performed for the B/Phuket/3073/2013 virus as previously described (61).

### FcγRIIIa dimer ELISA.

The capacity of anti-influenza antibodies in Flu-IVIG and patient sera samples to bind human FcγRs was assessed using a recombinant soluble human FcγRIIIa dimer ELISA, as previously described (53, 59). The recombinant soluble human FcγRIIIa and FcγRIIa dimers were provided by P. Mark Hogarth and Bruce Wines (Burnet Institute, Melbourne, Australia). Briefly, 50 ng of commercially sourced recombinant HA (from A/Cali09 or B/Phuket), NA (from B/Phuket), or SIV gp120 was coated in the wells of 96-well NUNC Maxisorb plates (Thermo Fisher Scientific) overnight at 4°C. The wells of the plates were blocked with 1% bovine serum albumin (BSA; Sigma-Aldrich) and 1 mM EDTA (Sigma-Aldrich) in PBS (PBSE/BSA) for 1 hour at 37°C. Two-fold serial dilutions of patient sera (starting at 1:40 dilution) were added to the wells and incubated for 1 hour at 37°C. Plates were washed prior to incubation with 50 μL of 0.1 μg/mL biotinylated human FcγRIIIa dimer for 1 hour at 37°C. After washing, plates were incubated with a 1:10,000 dilution of Pierce High Sensitivity HRP-Streptavidin (Thermo Fisher Scientific) for 1 hour at 37°C and then washed. Color was developed by adding 3,3′,5,5′-tetramethylbenzidine (TMB; Sigma-Aldrich) and then stopped with 1 M HCl, and absorbance read at 450 nm. Wells coated with SIV gp120 and incubated with Flu-IVIG or patient sera were used as negative control wells to detect nonspecific or background binding. Endpoint titers were calculated using 3 times the background binding value in antigen-coated wells without sera.

### Luminex bead-based multiplex assay.

A multiplex assay to detect influenza virus–specific antibodies was performed as previously described (43, 62, 63). Bio-Plex Pro Magnetic COOH bead sets (Bio-Rad Laboratories), each with different fluorescent properties, were coupled to 10 μg of A/Cali09 HA, B/Phuket HA, B/Phuket NA, or SIV gp120 (as a negative control) using 1.25 million beads per antigen. Covalent coupling of the antigens to the magnetic carboxylated beads was performed using a 2-step carbodiimide reaction (43, 62, 63).

A working bead mixture, containing 1000 of each bead type per well, was combined with 50 μL of a 1:100 dilution of patient sera into the wells of a black, clear-bottom, 96-well plate (Greiner Bio-One). Mouse anti–human IgG1–IgG4 (clone hinge-PE 4E3, Fc-PE HP6002, hinge-PE HP6050, and Fc-PE HP6025) and IgA1 and IgA2 antibodies (clone PE B3506B4 and PE A9604D2) conjugated to R-phycoerythrin (PE; Southern Biotech) were added at 1.3 μg/mL in 50 μL per well to detect antigen-specific IgG and IgA bound to fluorescent beads. All plates were washed manually using a magnetic plate separator (Luminex) and read on the Bio-Plex MAGPIX Multiplex reader or Luminex 200. Binding of the PE-conjugated detector antibodies was measured to calculate median fluorescence intensity (MFI). Double background subtraction was performed for each well, first subtracting blank wells (sheath only) followed by subtraction of the SIV gp120–coupled bead signal (background or nonspecific binding). To measure the capacity of influenza-specific IgG to cross-link human FcγRs, 50 μL of biotinylated recombinant soluble human FcγRIIa or FcγRIIIa dimer was added to the wells at a concentration of 1.3 μg/mL followed by washing and the addition of 50 μL of streptavidin PE (SAPE; Invitrogen) at 1 μg/mL to the wells. The plates were then incubated for 2 hours on a plate shaker at room temperature, washed, and read as described above.

The concentrations of IL-1β, IL-6, TNF-α, and IFN-γ were measured in participant sera using the ProcartaPlex 4-plex custom cytokine assay (custom assay ID MXWCX9T; Thermo Fisher Scientific), as described in the manufacturer’s protocol. Cytokine concentrations were calculated by the ProcartaPlex Analysis App (Thermo Fisher Scientific) using a standard curve derived from known reference concentrations of each cytokine provided by the manufacturer. A 5-parameter logistic (5PL) curve fit model was applied to the standard curve for analysis.

### Antibody-mediated NK cell activation assay.

To assess antibody-dependent NK cell activation, expression of the degranulation marker CD107a by an NK-92-FcγRIIIa-GFP cell line was measured in response to antibodies immobilized by plate-bound influenza proteins, as previously described (33, 53). The NK-92-FcγRIIIa-GFP cell line was provided by Kerry Campbell (Institute of Cancer Research, Philadelphia, Pennsylvania, USA). Briefly, 96-well NUNC Maxisorb plates (Thermo Fisher Scientific) were coated with 600 ng of influenza HA (from A/Cali09 or B/Phuket) or SIV gp120 (SinoBiological) and incubated with a 1:80 dilution of patient sera for 2 hours at 37°C. Next, 2 × 10^5^ NK-92-FcγRIIIa-GFP cells (expressing the V176 variant of FcγRIIIa conjugated to GFP, provided by K. Campbell, Institute for Cancer Research) were added for 5 hours at 37°C. Then, the NK-92-FcγRIIIa-GFP cells were then incubated with 1 mM EDTA and anti-CD107a APC-Cy7 (clone H4A3; BioLegend) for 30 minutes in the dark. Cells were washed twice, fixed with formaldehyde, and acquired on a FACSCanto flow cytometer (Becton Dickinson). The proportion of NK-92-FcγRIIIa-GFP^+^ cells expressing CD107a was quantified. Wells coated with SIV gp120 and incubated with patient sera were used as negative control wells to detect nonspecific or background binding. For each patient sample, the percentage of CD107a^+^ NK-92 cells in the SIV gp120 negative control wells was subtracted from the percentage of CD107a^+^ NK-92 cells in the HA-coated wells.

### Statistics.

Analysis of covariance, with the preinfusion or d0 level as a covariate, was used to compare differences between the placebo and Flu-IVIG treatment groups on d1 after infusion for HAI titer, FcγRIIIa-binding antibody titer, FcγRIIIa-binding antibody MFI, FcγRIIa-binding antibody MFI, pan IgG MFI, and IgG1 MFI. The *P* value for these comparisons represents the difference between treatment groups for log_2_(d1 postinfusion antibody level), controlling for preinfusion (d0) level.

The association between Flu-IVIG treatment and changes in HA- and NA-specific antibody MFIs for IgG2, IgG3, and IgG4 were investigated using Tobit regression models in B/Yamagata- and A/H1N1-infected participants. The Tobit regression models left censored the “zeros” values (which represent MFIs at or below background levels) of the immunological parameters for IgG2, IgG3, and IgG4 antibodies. Given that antibody MFI values were transformed to the log_2_ scale, the results from the Tobit models are presented as exponentiated coefficients and interpreted as relative mean differences of Flu-IVIG group compared with the placebo group, after controlling for baseline or day 0 (d0) preinfusion antibody levels. Of note, the “zero” values of the immune markers were not log_2_-transformed when left censoring them in the Tobit regression models. A sensitivity analysis that replaced “zero” values with log_2_-transformed value of 0.001 for the left censoring in the models showed similar results. *P* values less than 0.05 were considered statistically significant. All analyses were conducted in Stata version 16.1 (StataCorp Inc.).

On d3, d5, and d7, the treatment effect was estimated for subgroups defined by infection with B/Yamagata versus A/H1N1 using a proportional odds logistic model to estimate a summary OR for being in a better category. Analyses were adjusted for baseline ordinal category and region of enrolment. We analyzed heterogeneity of the treatment effect by adding an interaction term to the models. Similar models, without the described baseline adjustments, were also used to assess the treatment effect across subgroups defined by median A/Cali HA- and B/Phuket HA-specific FcγRIIIa dimer–binding antibody titer levels at baseline.

The associations between d3, d5, and d7 ordinal outcomes and antibody titers (IgG1, IgG2, IgG3, IgG4, IgA1, IgA2, FcγR2a, FcγR3a, NKCAA, HAI), at baseline/d0 before infusion and d1 after infusion were investigated using proportional odds logistic regression to estimate a summary OR for being in a better category in both B/Yamagata- and A/H1N1-infected participants. The multivariate proportional odds logistic models were adjusted for risk score on day 7 (for details see ref. 8), baseline antibody level (for models of d1 antibody levels), and treatment group (IVIG/placebo).

### Study approval.

The INSIGHT 006: Flu-IVIG trial (NCT02287467) was an international, double-blinded, placebo-controlled clinical trial designed and conducted by the International Network for Strategic Initiatives in Global HIV trials (INSIGHT). This trial included 45 hospitals in Argentina, Australia, Demark, Greece, Mexico, Spain, Thailand, the United Kingdom, and the United States. All patients provided written informed consent and this trial was approved by the institutional ethics committee or review board at each clinical site.

## Author contributions

HAV performed the experimental work, data interpretation, and figure preparation. HAV, SJK, and JDN contributed to study design. DNW, WMH, MGL, and JDN were involved in data analysis and interpretation. HP and IGB performed the HAI assays. RTD, DED, MKJ, BA, CTB, and AM were involved in sample collection and the design of the INSIGHT 006: FLU-IVIG clinical trial. JHB played a critical role in obtaining and overseeing the Flu-IVIG product. HAV and SJK conceived the study and wrote the manuscript. All authors contributed to preparation of the manuscript.

## Supplementary Material

Supplemental data

ICMJE disclosure forms

## Figures and Tables

**Table 1:**
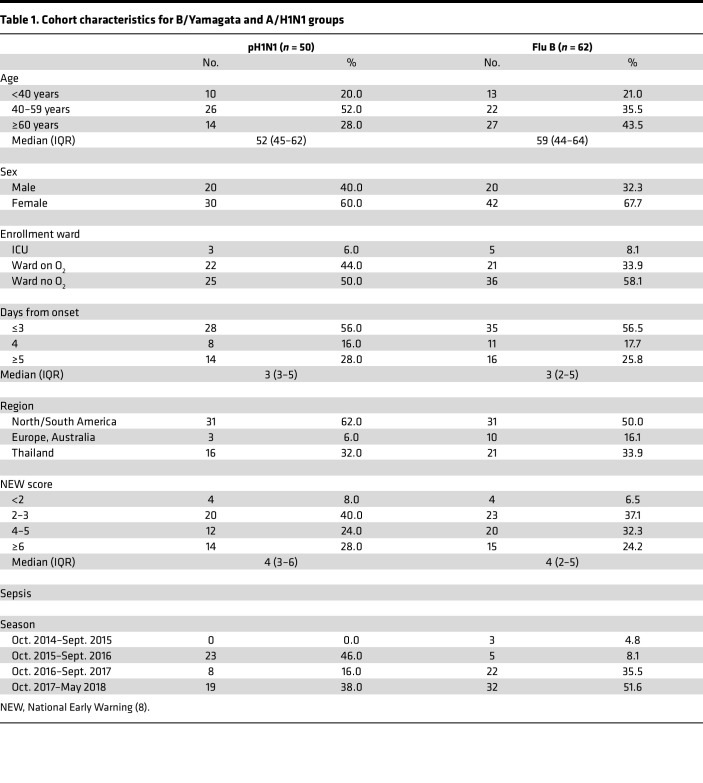
Cohort characteristics for B/Yamagata and A/H1N1 groups

## Version 1 06/08/2023

In-Press Preview

## Version 2 07/24/2023

Electronic publication
