# Supplementary material for: Understanding the treatment benefit of hyperimmune anti-influenza intravenous immunoglobulin (Flu-IVIG) for severe human influenza
Source: JCI Insight. 2023 Jul 24;8(14):e167464. doi: 10.1172/jci.insight.167464 (PMC10443807; doi:10.1172/jci.insight.167464)
Supplement: Supplemental data [file jciinsight-8-167464-s178.pdf]

<u>**Supplemental Material**</u>

Understanding the treatment benefit of hyperimmune anti-influenza intravenous immunoglobulin (Flu-IVIG) for severe human influenza

Hillary A Vanderven[1-3*], Deborah N Wentworth[4], Win Min Han[5], Heidi Peck[6], Ian Barr[6], Richard T Davey, Jr.[7], John Beigel[7], Dominic Dwyer[8], Mamta Jain[9], Brian Angus[10], Christian Brandt[11], Analia Mykietiuk[12], Matthew G Law[5], Jim D Neaton[4], and Stephen J Kent[3,12*] for the INSIGHT FLU-IVIG Study Group

[1]Biomedicine, College of Public Health, Medical and Veterinary Sciences, James Cook University, Douglas, Queensland, Australia;
[2]Australian Institute of Tropical Health and Medicine, James Cook University, Douglas, Queensland, Australia;
[3]Department of Microbiology and Immunology, Peter Doherty Institute for Infection and Immunity, University of Melbourne, Parkville, Victoria, Australia;
[4]Divison of Biostatistics, School of Public Health, University of Minnesota, Minneapolis, Minnesota, USA;
[5]The Kirby Institute for Infection and Immunity, University of New South Wales, Sydney, New South Wales, Australia;
[6]WHO Collaborating Centre for Reference and Research on Influenza at the Peter Doherty Institute of Infection and Immunity, Melbourne, Victoria, Australia;
[7]National Institute of Allergy and Infectious Disease (NIAID), Bethesda, Maryland, USA;
[8]New South Wales Health Pathology-Institute of Clinical Pathology and Medical Research, Westmead Hospital, Westmead, Australia;
[9]University of Texas Southwestern Medical Center, Dallas, Texas, USA;
[10]Nuttfield Department of Medicine, Oxford University, Oxford, OX39DU, United Kingdom;
[11]Sjællands Universitets Hospital, Roskilde, Denmark;
[12]Instituto Médico Platense, Buenos Aires, Argentina;
[13]Melbourne Sexual Health Centre and Department of Infectious Diseases, Alfred Health, Central Clinical School, Monash University, Carlton, Victoria, Australia.

Supplemental Acknowledgements

The writing group for this manuscript was: Vanderven HA, Wentworth DN, Han WN, Peck H, Barr I, Davey RT, Beigel J, Dwyer D, Jain M, Angus B, Brandt C, Mykietiuk A, Law MG, Neaton JD and SJ Kent

Other members of the INSIGHT FLU-IVIG Study Group who contributed to conducting the INSIGHT 006 FLU-IVIG clinical trial are listed below.

INSIGHT Statistical and Data Management Center: Jessica Butts, Eileen Denning, Alain DuChene, Eric Krum, Merrie Harrison, Sue Meger, Ross Peterson, Kien Quan, Megan Shaughnessy, Greg Thompson, David Vock

National Institute of Allergy and Infectious Diseases: Julia Metcalf

Leidos: Robin Dewar, Tauseef Rehman, Ven Natarajan, Rose McConnell

Advanced BioMedical Laboratories: Emily Flowers, Kenny Smith, Marie Hoover

Center for Biologics Evaluation and Research (CBER), U.S. Food and Drug Administration (FDA): Elizabeth M. Coyle

INSIGHT Community Advisory Board Representative: David Munroe

INSIGHT International Coordinating Centers

Copenhagen: Bitten Aagaard, Mary Pearson
London: Adam Cursley, Helen Webb, Fleur Hudson, Charlotte Russell, Aminata Sy, Cara Purvis, Brooke Jackson, Yolanda Collaco-Moraes.
Sydney: Dianne Carey, Rosemary Robson
Washington: Adriana Sánchez, Elizabeth Finley, Donna Conwell

INSIGHT Site Coordinating Centers

Argentina: Marcelo H. Losso, Luciana Gambardella, Cecilia Abela
Spain: Paco Lopez, Helena Alonso
Greece: Giota Touloumi, Vicky Gioukari, Olga Anagnostou
Thailand: Anchalee Avihingsanon, Kanitta Pussadee, Sasiwimol Ubolyam

INSIGHT Clinical Sites by Country

United States
Montefiore Medical Center: Bola Omotosho, Clemencia Solórzano
UT Southwestern Medical Center: Tianna Petersen, Kranthi Vysyaraju
Mayo Clinic: Stacey A. Rizza, Jennifer A Whitaker
Denver Public Health: Edward M. Gardner (PI), James A. Scott
Henry Ford Health System: Leslie Faber, Erica Pastor, Linda Makohon
Augusta University Research Institute, Inc: Rodger D. MacArthur (PI), L. Monique Hillman, Marti J. Farrough
Miami Valley Hospital: Hari M. Polenakovik (PI), Linda A. Clark, Roberto J. Colon
Minneapolis VA Health Care System, Pulmonary Section (n=4): Ken M. Kunisaki (PI), Miranda DeConcini, Susan A. Johnson
Duke University: Cameron R. Wolfe (PI), Laura Mkumba, June Y. Carbonneau

University of Pittsburgh Medical Center: Alison Morris (PI), Meghan E. Fitzpatrick, Cathy J. Kessinger
Case Western Reserve University: Robert A. Salata (PI), Karen A. Arters, Catherine M. Tasi
Cincinnati VA Medical Center: Ralph J. Panos(PI), Laura A. Lach
Cornell Clinical Trials Unit: Marshall J. Glesby (PI), Kirsis A. Ham, Valery G. Hughes
UCSD Antiviral Research Center: Robert T. Schooley (PI), Daniel Crouch, Leticia Muttera
University of Illinois at Chicago: Richard M. Novak (PI), Susan C. Bleasdale, Ariel E. Zuckerman

Thailand

Bamrasnaradura Infectious Diseases Institute: Weerawat Manosuthi (PI), Supeda Thaonyen, Thaniya Chiewcharn
Chulalongkorn University and HIV-NAT: Gompol Suwanpimolkul (PI), Sivaporn Gatechumpol, Sirikunya Bunpasang

United Kingdom

Oxford University Hospitals NHS Foundation Trust: Brian J. Angus (PI), Monique Anderson, Marcus Morgan
St James' University Hospital: Jane Minton (PI), Maria N. Gkamaletsou, Joe Hambleton
Newcastle upon Tyne Hospitals NHS Foundation Trust: David A. Price (PI)
Royal Sussex County Hospital: Martin J. Llewelyn (PI), Jonathan Sweetman

Spain

Hospital General Universitario Gregorio Marañón: Javier Carbone
Hospital Universitario La Paz: Jose R. Arribas (PI), Rocio Montejano
Hospital Universitario de Álava: Jose L. Lobo Beristain (PI), Iñaki Z. Martinez
Hospital Universitario HM Montepríncipe: Jose Barberan (PI), Paola Hernandez

Australia

Westmead Hospital: Dominic E. Dwyer (PI), Jen Kok

Denmark

CHIP, Department of Infectious Diseases, Section 2100: Alvaro Borges (PI), Christian T. Brandt, Lene S. Knudsen

Greece

AIDS Unit, Academic Dept of Pathophysiology of Athens Medical School “Laikon” Athens General Hospital: Nikolaos Sypsas (PI), Costas Constantinou, Antonios Markogiannakis
1st Department of Critical Care and Pulmonary Medicine, University of Athens School of Medicine, Evangelismos Hospital: Spyros Zakynthinos (PI), Paraskevi Katsaounou, Ioannis Kalomenidis

Argentina

Instituto Médico Platense: Analia Mykietiuk (PI), Maria F. Alzogaray, Mora Obed
Hospital General de Agudos JM Ramos Mejia: Laura M. Macias, Juan Ebensrtejin, Patricia Burgoa
Sanatorio Británico S.A.: Esteban Nannini (PI), Matias Lahitte

Mexico

Instituto Nacional de Ciencias Médicas y Nutrición Salvador Zubirán: Santiago Perez-Patrigeon (PI), José Arturo Martínez-Orozco, Juan Pablo Ramírez-Hinojosa

Data and Safety Monitoring Board (DSMB)

An independent DSMB had complete access to unblinded data during the trial’s conduct and was responsible for periodic review of safety and efficacy. Members of the DSMB were: William Blackwelder (chair), David Parenti, Mary Young, Wilbur Chen, Larry Moulton, and Nikhil Hirani.

Supplemental Figure 1

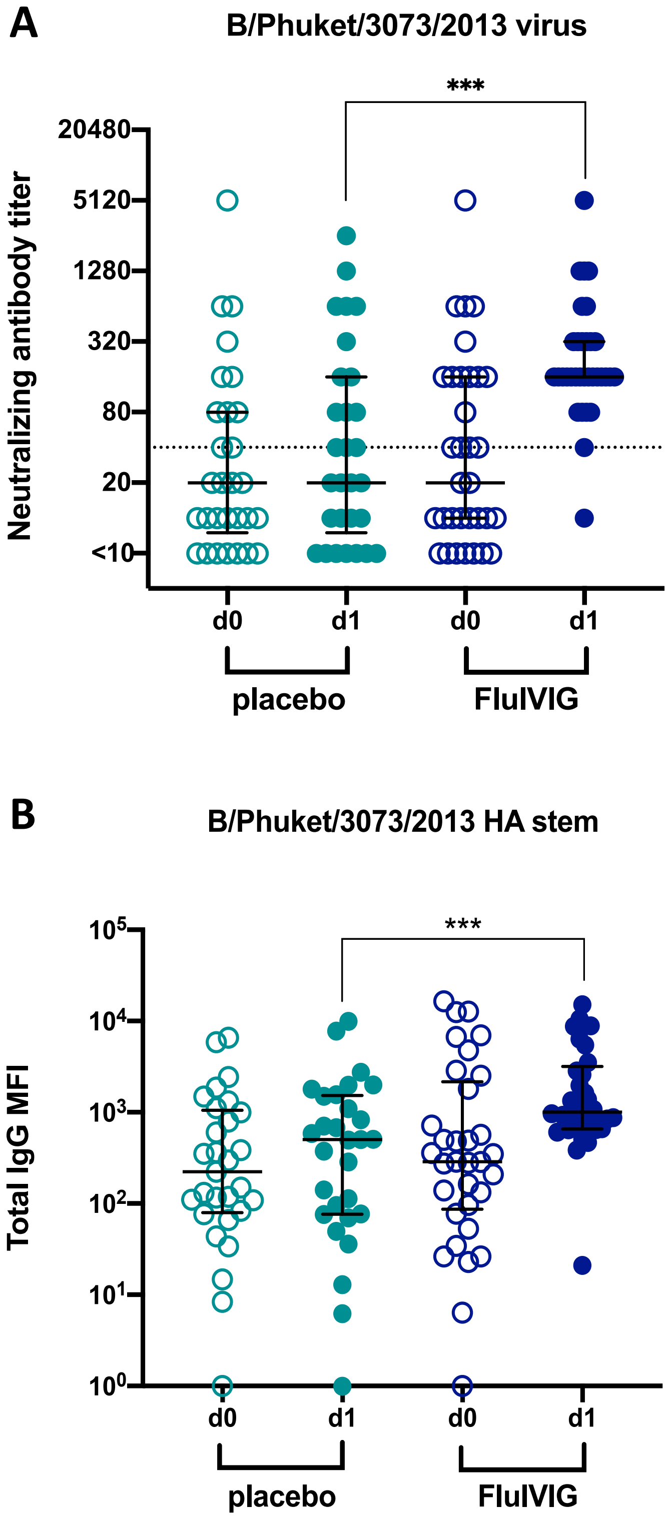

Supplemental Figure 1. Neutralizing antibody titers and HA stem-specific IgG day 1 post-infusion with influenza-specific hyperimmune immunoglobulin (Flu-IVIG) in patients hospitalised with influenza B. Pre-infusion (d0; open symbols) and post-infusion (d1; closed symbols) median neutralizing antibody titer (with interquartile range) by microneutralization assay (MNA) against the B/Phuket/3073/2013 virus (A) and median fluorescence intensity (MFI; with interquartile range) of total IgG against the B/Phuket/3073/2013 HA stem (B) are shown for the placebo (n = 29) or Flu-IVIG (n = 33) infused B/Yamagata patients. Analysis of covariance, with the pre-infusion or d0 level as a covariate, was used to compare differences between the placebo and Flu-IVIG treatment groups at d1 post-infusion. The p-value represents the difference between treatment groups for log_2 d1 post-infusion titer or MFI controlling for pre-infusion (d0) titer or MFI. *** $P < 0.001$

Supplemental Figure 2

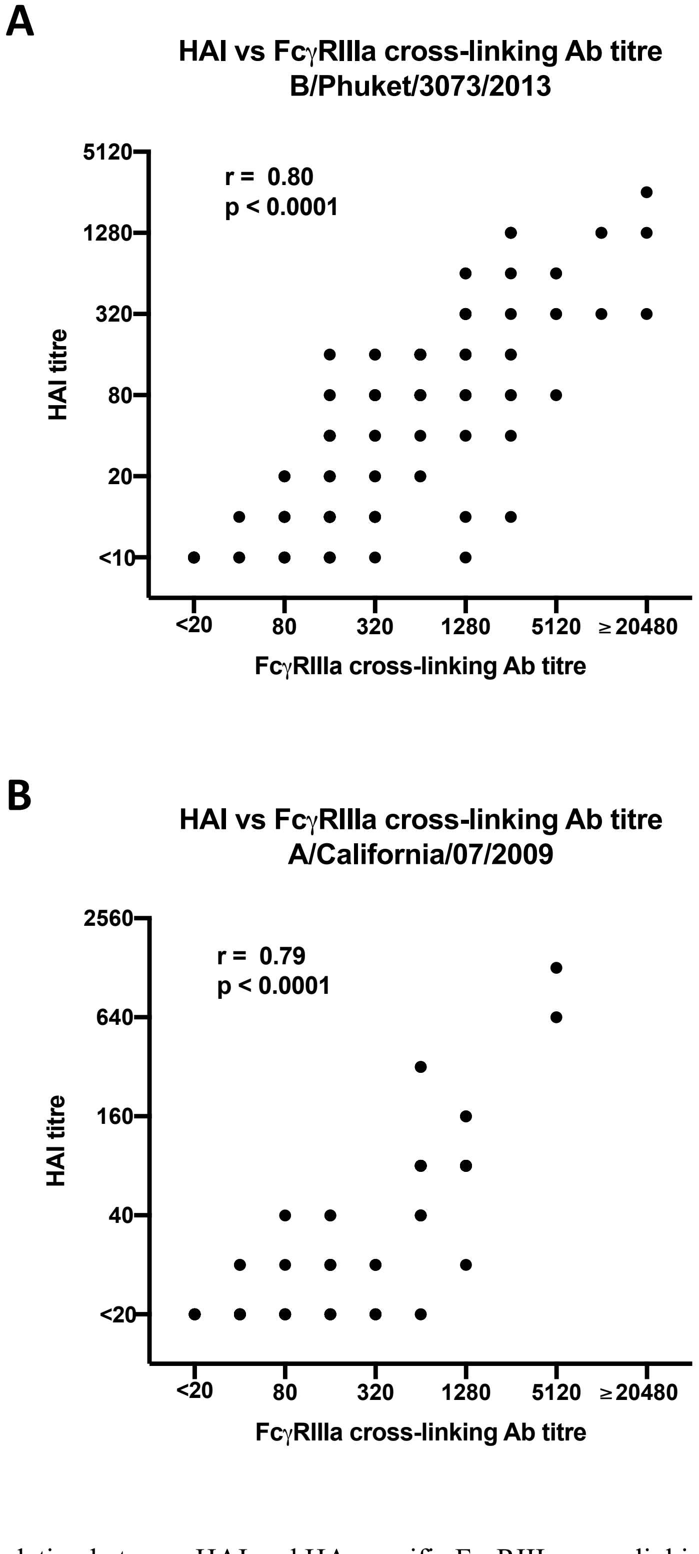

Supplemental Figure 2. Correlation between HAI and HA-specific FcγRIIIa cross-linking antibody titer in B/Yamagata and A/H1N1 infected patients treated with Flu-IVIG or placebo. Spearman correlations between HAI and HA-specific FcγRIIIa cross-linking antibody titers in pre-infusion (d0) and day 1 (d1) post-infusion serum samples following treatment with Flu-IVIG or placebo in B/Yamagata (n = 62; A) and A/H1N1 (n = 50; B) infected patients are shown. Some points represent multiple superimposed patient samples, who have identical HAI and FcγRIIIa cross-linking antibody titers.

Supplemental Figure 3

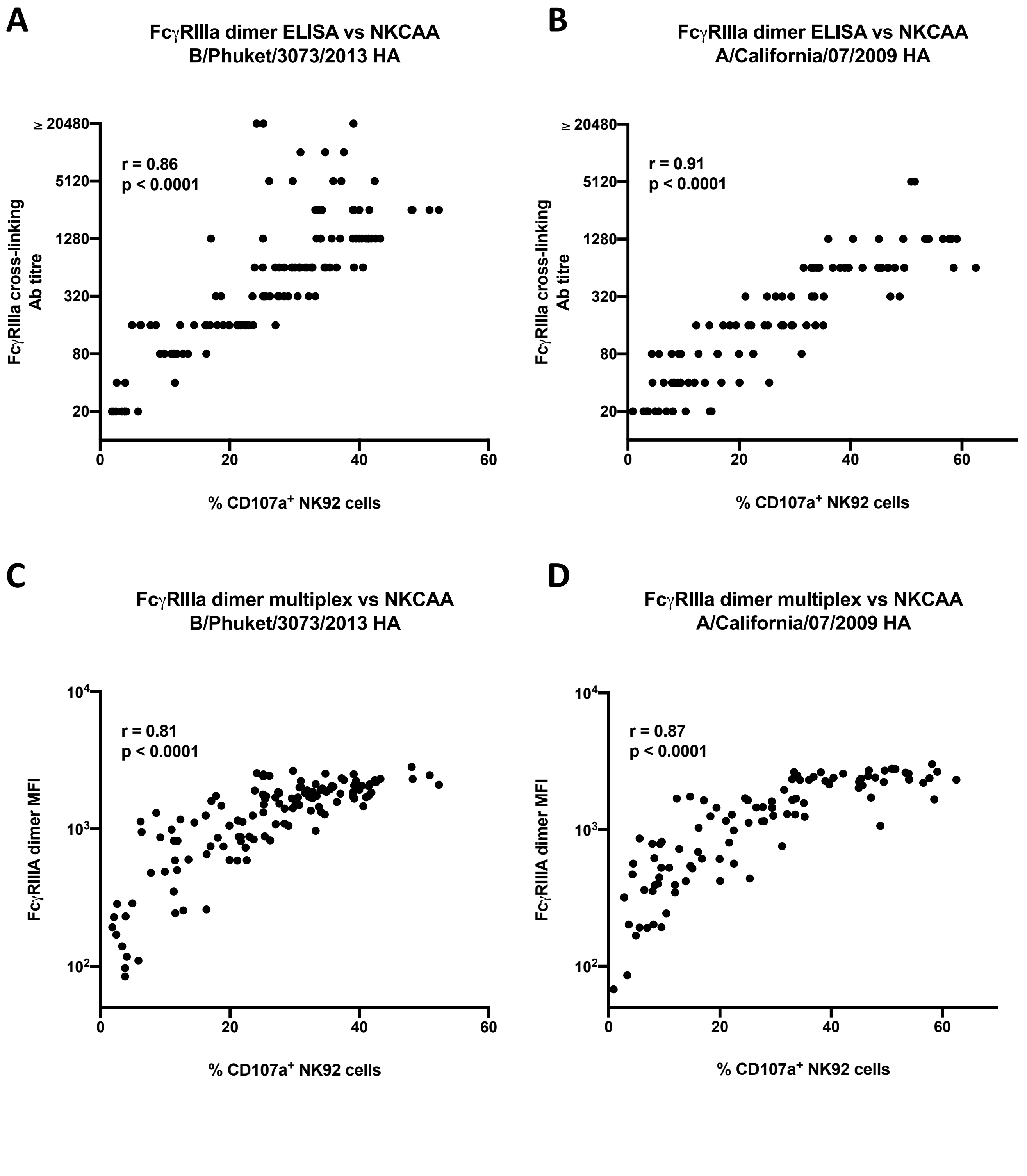

Supplemental Figure 3. Correlation between HA-specific FcγRIIIa cross-linking antibody and NK cell activation in B/Yamagata and A/H1N1 infected patients treated with Flu-IVIG or placebo. Spearman correlations are shown between NK cell activation (%CD107a+ NK-92-FcγRIIIa-GFP cells) and HA-specific FcγRIIIa cross-linking antibody titers detected by ELISA in pre- and day 1 (d1) post-infusion serum samples following treatment with Flu-IVIG or placebo in B/Yamagata (n = 62; A) and A/H1N1 (n = 50; B) infected patients. Spearman correlations between NK cell activation and median fluorescence intensity (MFI) of HA-specific FcγRIIIa cross-linking antibody detected by bead-based multiplex pre- and day 1 (d1) post-infusion serum samples following treatment with Flu-IVIG or placebo in B/Yamagata (n = 62; C) and A/H1N1 (n = 50; D) infected patients are also shown.

Supplemental Figure 4

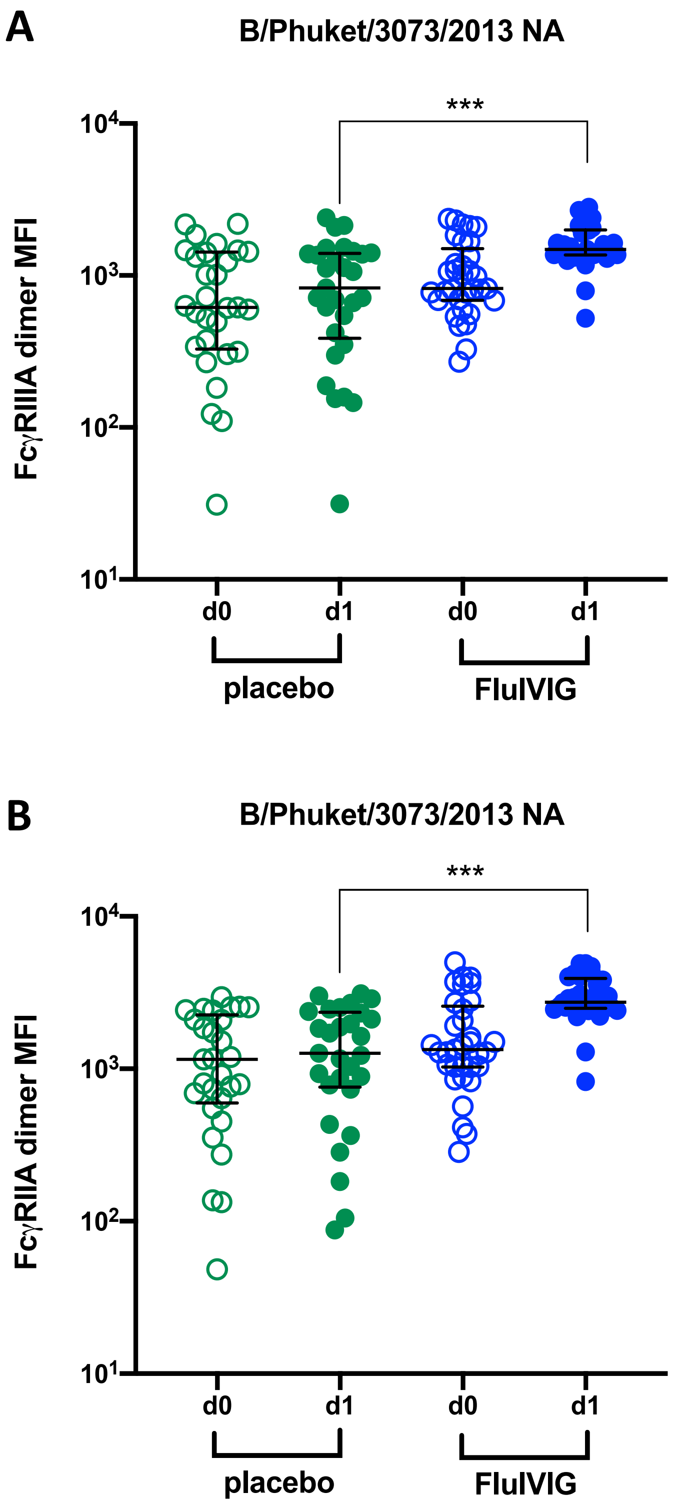

Supplemental Figure 4. NA-specific FcγR binding day 1 post-infusion with Flu-IVIG in patients hospitalised with severe influenza B. A bead-based FcγR dimer multiplex was used to examine the median fluorescence intensity (MFI) of NA-specific FcγRIIIa and FcγRIIa binding antibody in patient sera. Pre-infusion (d0; open symbols) and post-infusion (d1; closed symbols) FcγRIIIa dimer binding (A) and FcγRIIa dimer binding (B) median MFI with interquartile range against the B/Phuket/3073/2013 NA were measured for the placebo (n = 29) or Flu-IVIG (n = 33) treated B/Yamagata infected patients. Analysis of covariance, with the pre-infusion or d0 level as a covariate, was used to compare differences between the placebo and Flu-IVIG treatment groups at d1 post-infusion. The p-value represents the difference between treatment groups for $\log_2$ d1 post-infusion controlling for pre-infusion (d) MFI. *** $P < 0.001$

Supplemental Figure 5

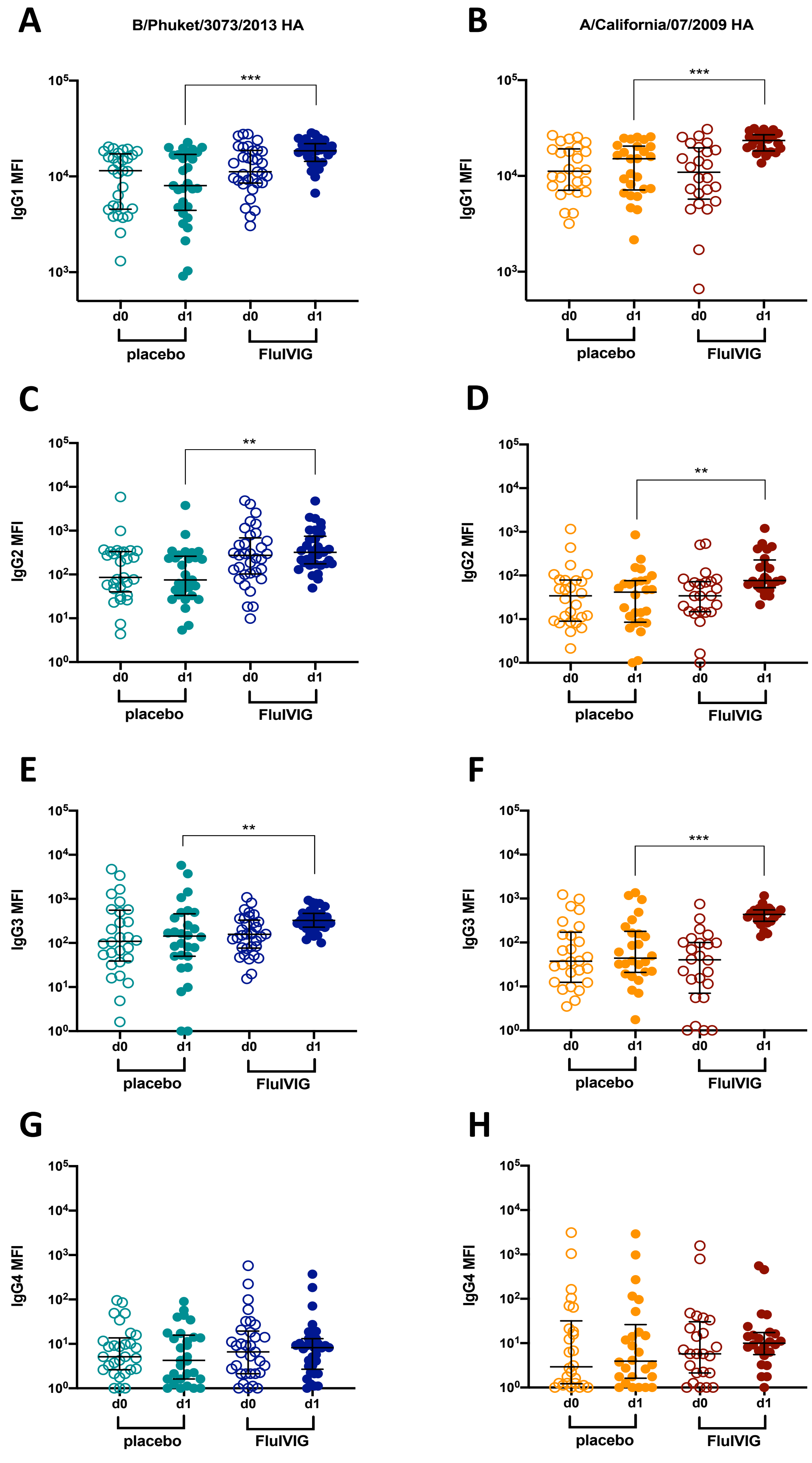

Supplemental Figure 5. HA-specific IgG subclasses following treatment with Flu-IVIG in patients hospitalised with severe influenza. A bead-based IgG subclass multiplex was used to determine the median fluorescence intensity (MFI) of HA-specific IgG subclasses in patient sera. Pre-infusion (d0; open symbols) and post-infusion (d1; closed symbols) median MFI with interquartile range for IgG1 (A & B), IgG2 (C & D), IgG3 (E & F) and IgG4 (G & H) against the B/Phuket/3073/2013 HA (left figure panels) or the A/California/07/2009(H1N1) HA (right figure panels) were measured for the placebo or Flu-IVIG treated B/Yamagata (Flu-IVIG n = 33 and placebo n = 29) and A/H1N1 (Flu-IVIG n = 24 and placebo n = 26) infected patients, respectively. Analysis of covariance, with the pre-infusion or d0 level as a covariate, was used to compare differences between the placebo and Flu-IVIG treatment groups at d1 post-infusion. The p-value represents the difference between treatment groups for $\log_2$ d1 post-infusion controlling for pre-infusion (d1) MFI. ** $P < 0.01$, *** $P < 0.001$

Supplemental Figure 6

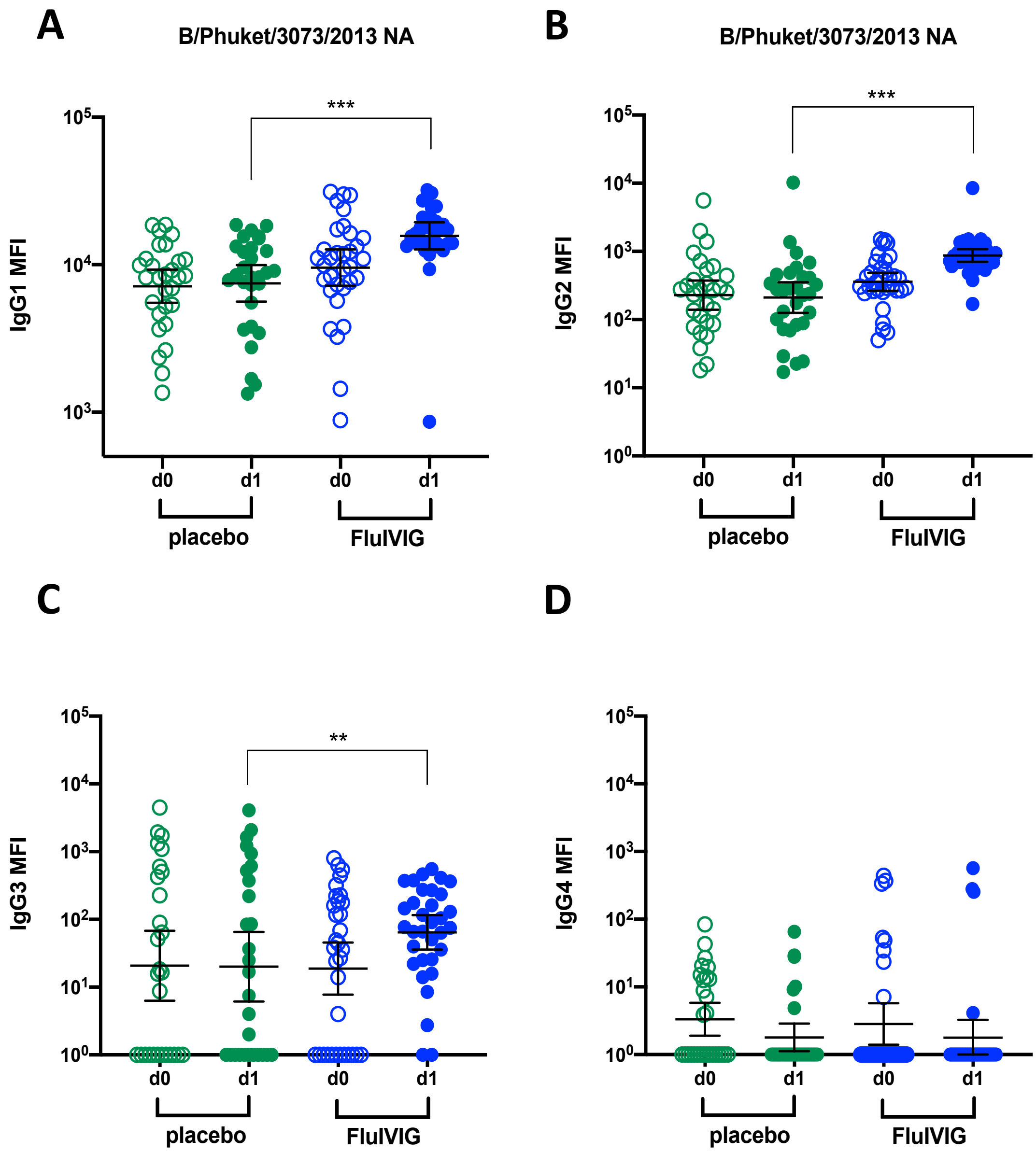

Supplemental Figure 6. NA-specific IgG subclasses day 1 post-infusion with Flu-IVIG in patients hospitalised with severe influenza B. A bead-based IgG subclass multiplex was used to examine the median fluorescence intensity (MFI) of NA-specific IgG subclasses in patient sera. Pre-infusion (d0; open symbols) and post-infusion (d1; closed symbols) median MFI with interquartile range for IgG1 (A), IgG2 (B), IgG3 (C) and IgG4 (D) against the B/Phuket/3073/2013 NA were measured for the placebo (n = 29) or Flu-IVIG (n = 33) treated B/Yamagata infected patients. Analysis of covariance, with the pre-infusion or d0 level as a covariate, was used to compare differences between the placebo and Flu-IVIG treatment groups at d1 post-infusion. The p-value represents the difference between treatment groups for log_2 d1 post-infusion controlling for pre-infusion (d0) MFI. ** P <0.01, *** P < 0.001

Supplemental Figure 7

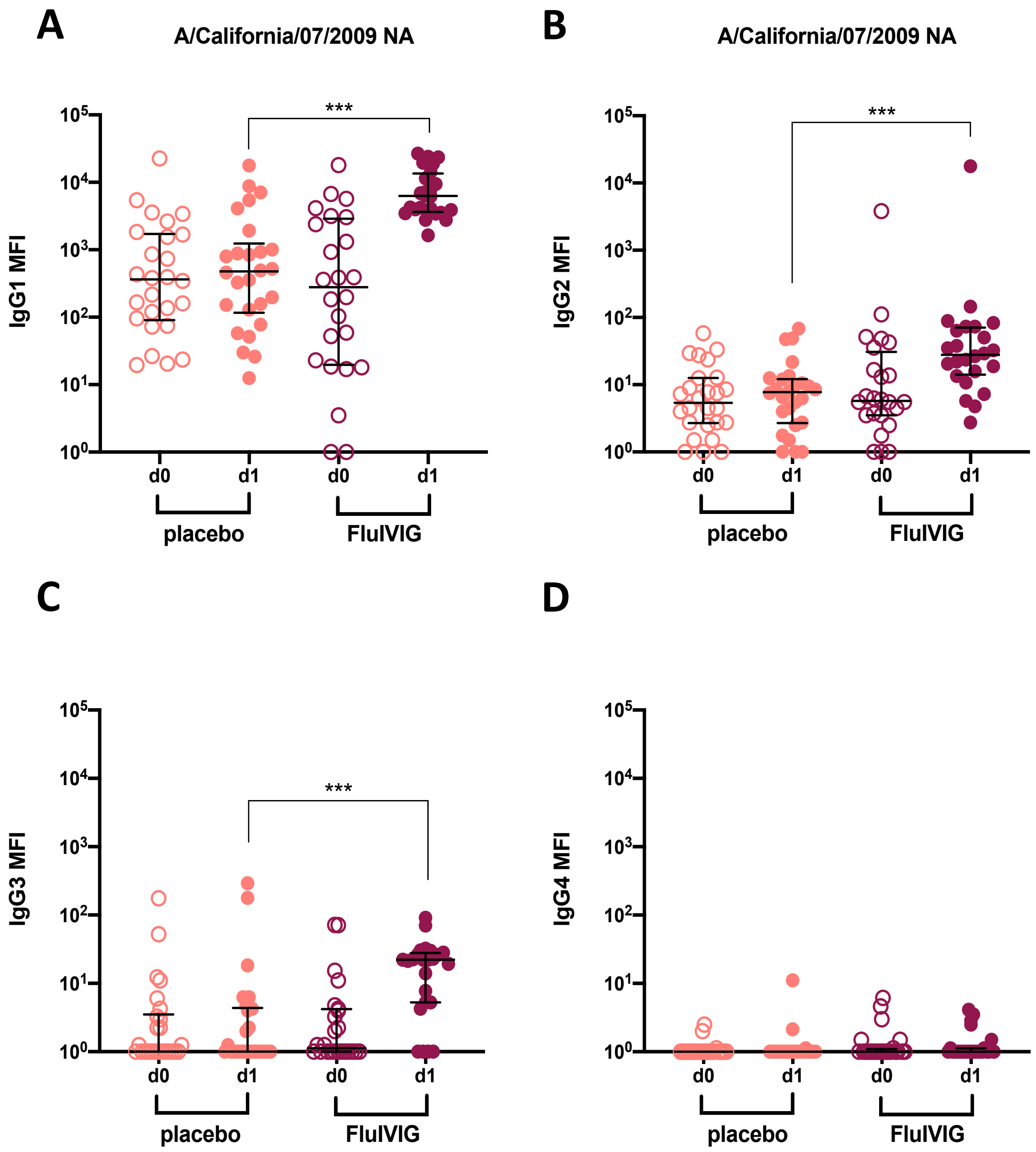

Supplemental Figure 7. NA-specific IgG subclasses day 1 post-infusion with Flu-IVIG in patients hospitalised with severe influenza A. A bead-based IgG subclass multiplex was used to examine the median fluorescence intensity (MFI) of NA-specific IgG subclasses in patient sera. Pre-infusion (d0; open symbols) and post-infusion (d1; closed symbols) median MFI with interquartile range for IgG1 (A), IgG2 (B), IgG3 (C) and IgG4 (D) against the A/California/07/2009(H1N1) NA were measured for the placebo (n = 26) or Flu-IVIG (n = 24) treated A/H1N1 infected patients. Analysis of covariance, with the pre-infusion or d0 level as a covariate, was used to compare differences between the placebo and Flu-IVIG treatment groups at d1 post-infusion. The p-value represents the difference between treatment groups for $\log_2$ d1 post-infusion controlling for pre-infusion (d0) MFI. *** P < 0.001

Supplemental Figure 8

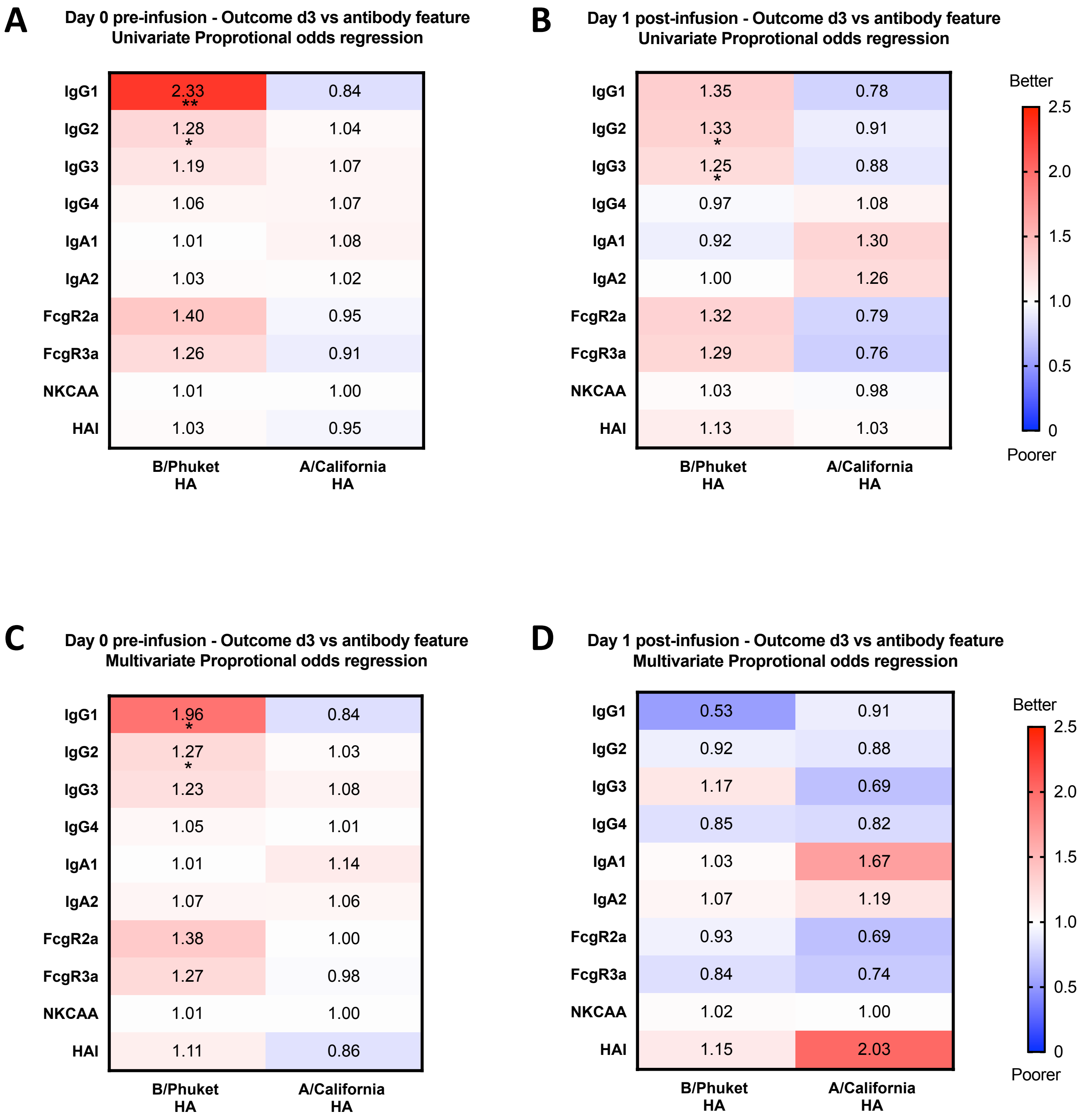

Supplemental Figure 8. Associations between influenza antibody features and clinical outcomes at day 3 post-infusion in patients hospitalised with severe B/Yamagata and A/H1N1 influenza. The association between antibody features and day 3 (d3) post-infusion ordinal outcomes were investigated using univariate and multivariate proportional odds regression models, with the multivariate model adjusting for baseline antibody level, treatment group (Flu-IVIG/placebo) and risk score at day 7. Heat maps show summary odds ratios (ORs) for patients hospitalised with B/Yamagata (n = 62; left side of heat map) and A/H1N1 (n = 50; right side of heat map) influenza at pre-infusion (A and C) and d1 post-infusion (B and D) timepoints generated using univariate (A and B) and multivariate (C and D) proportional odds regression models. ORs greater than one indicate that patients with higher antibody levels have improved odds of being in a better outcome category at d3 post-infusion and ORs less than one indicate that patients with lower antibody levels have improved odds of being in a better outcome category. * P <0.05, ** P <0.01

Supplemental Figure 9

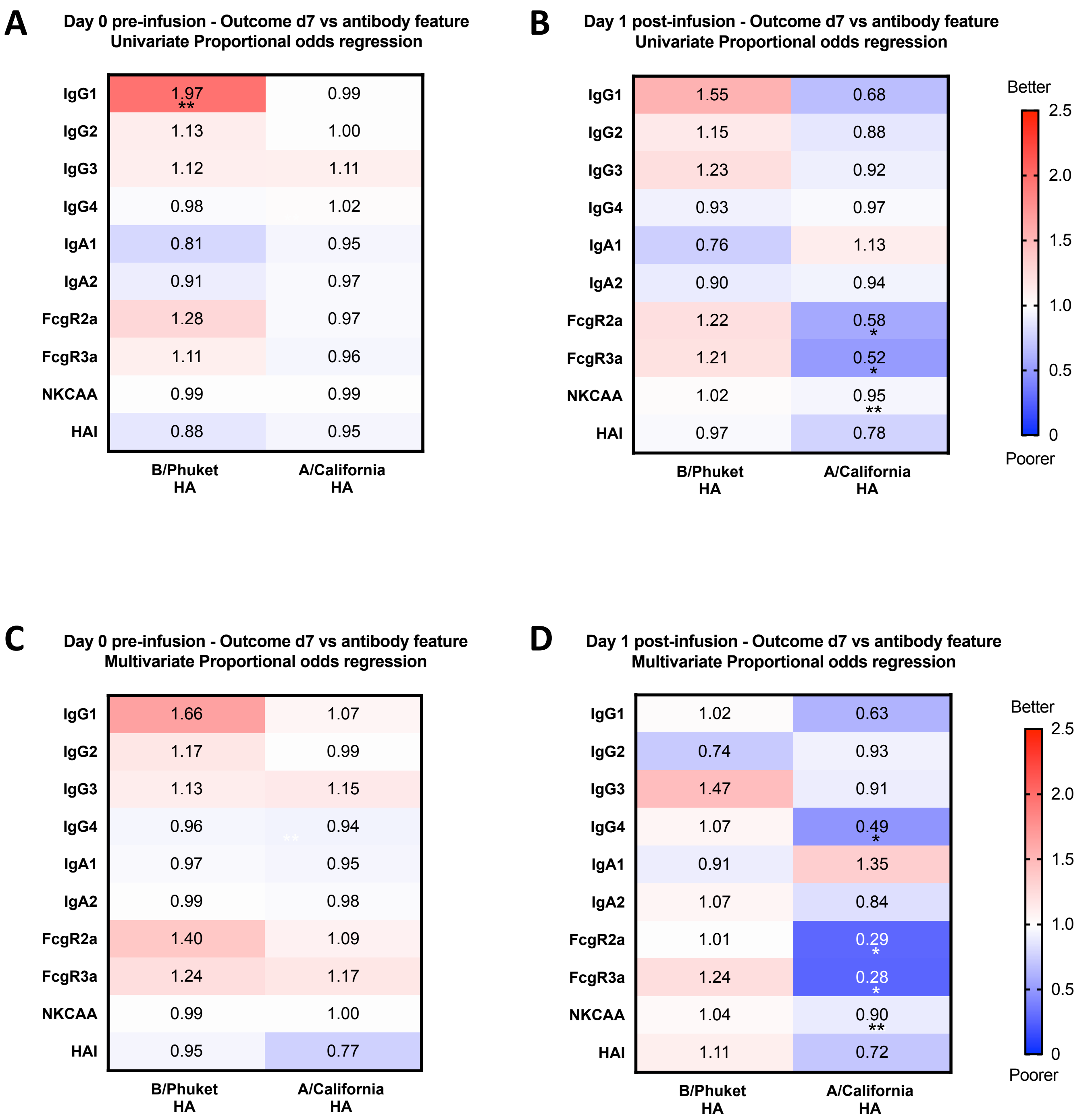

Supplemental Figure 9. Associations between influenza antibody features and clinical outcomes at day 7 post-infusion in patients hospitalised with severe B/Yamagata and A/H1N1 influenza. The association between antibody features and day 7 (d7) post-infusion ordinal outcomes were investigated using univariate and multivariate proportional odds regression models, with the multivariate model adjusting for baseline antibody level, treatment group (Flu-IVIG/placebo) and risk score at day 7. Heat maps show summary odds ratios (ORs) for patients hospitalised with B/Yamagata (n = 62; left side of heat map) and A/H1N1 (n = 50; right side of heat map) influenza at pre-infusion (A and C) and d1 post-infusion (B and D) timepoints generated using univariate (A and B) and multivariate (C and D) proportional odds regression models. ORs greater than one indicate that patients with higher antibody levels have improved odds of being in a better outcome category at d7 post-infusion and ORs less than one indicate that patients with lower antibody levels have improved odds of being in a better outcome category. * P <0.05, ** P <0.01

Supplemental Figure 10

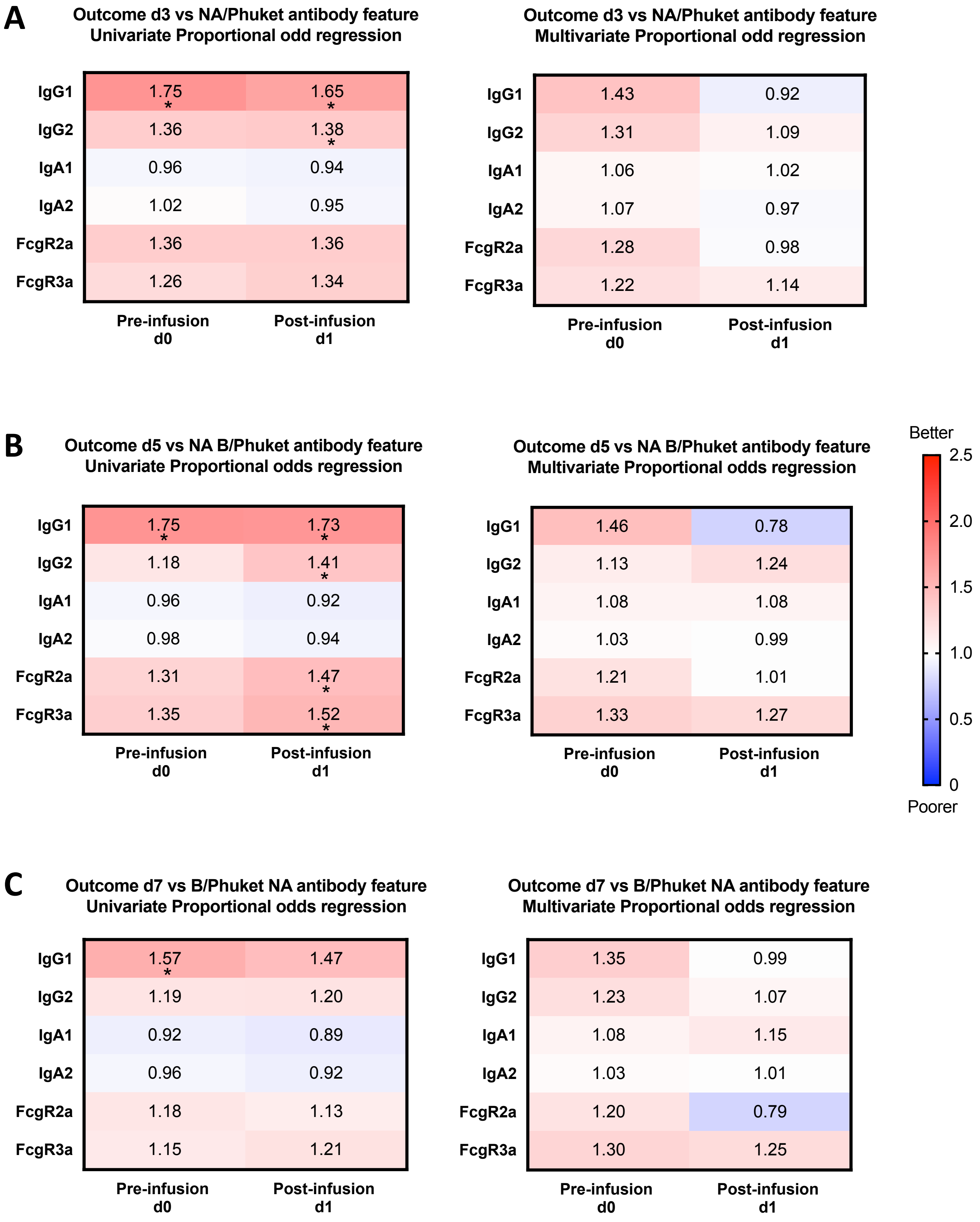

Supplemental Figure 10. Associations between NA-specific influenza antibody features and clinical outcomes in patients hospitalised with severe influenza B. The association between antibody features and (A) day 3 (d3), (B) day 5 (d5) and (C) day 7 (d7) post-infusion ordinal outcomes were investigated using univariate and multivariate proportional odds regression models, with the multivariate model adjusting for baseline antibody level, treatment group (Flu-IVIG/placebo) and risk score at day 7. Heat maps show summary odds ratios (ORs) for patients hospitalised with B/Yamagata influenza (n = 62) at pre-infusion (left side of heat map) and d1 post-infusion (right side of heat map) timepoints generated using univariate (right figure panel) and multivariate (left figure panel) proportional odds regression models. ORs greater than one indicate that patients with higher antibody levels have improved odds of being in a better outcome category at the post-infusion timepoint indicated, whereas ORs less than one indicate that patients with lower antibody levels have improved odds of being in a better outcome category. * $P < 0.05$

Supplemental Figure 11

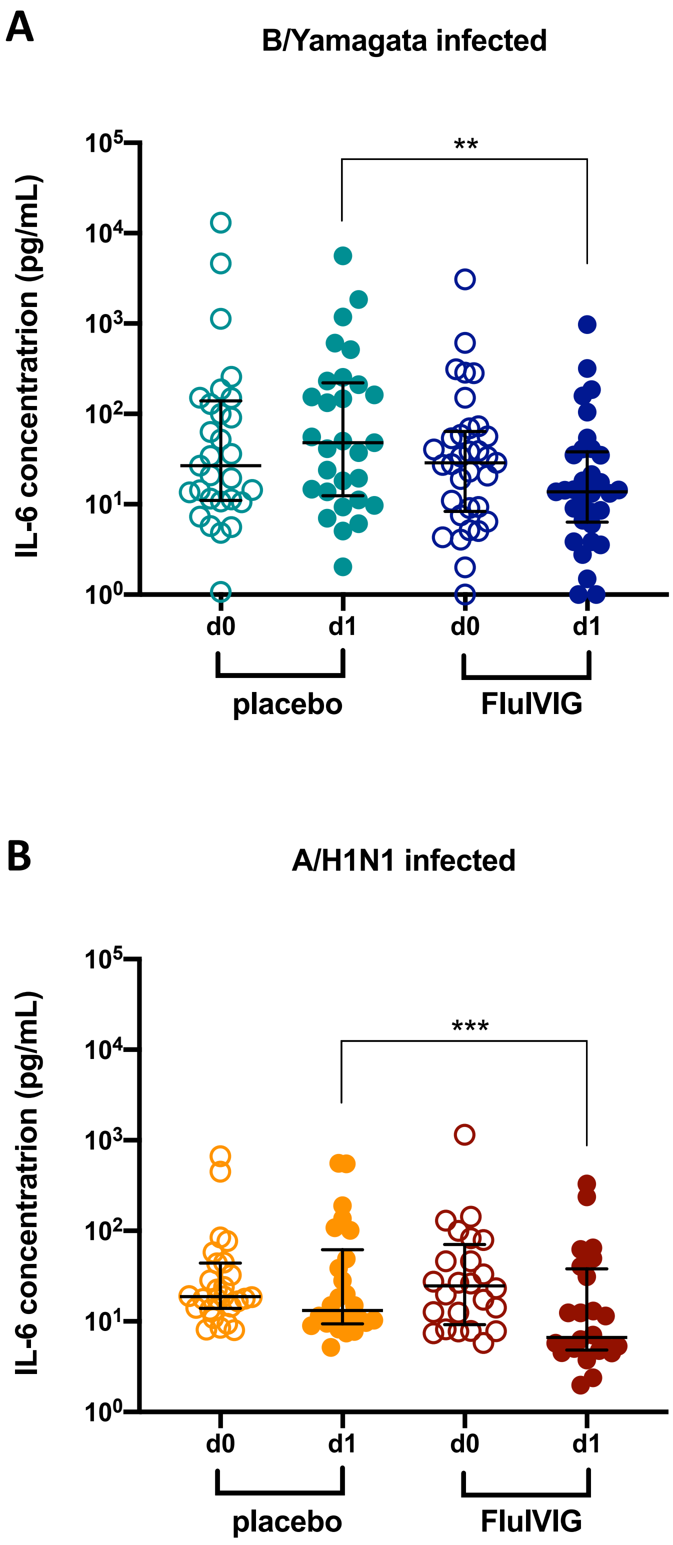

Supplemental Figure 11. Serum concentration of interleukin-6 (IL-6) day 1 post-infusion with Flu-IVIG in patients hospitalised with severe influenza. A bead-based multiplex was used to determine the concentration of IL-6 in patient sera. Pre-infusion (d0; open symbols) and post-infusion (d1; closed symbols) median concentrations of IL-6 with interquartile ranges were measured for the placebo or Flu-IVIG treated (A) B/Yamagata (Flu-IVIG n = 33 and placebo n = 29) and (B) A/H1N1 (Flu-IVIG n = 24 and placebo n = 26) infected patients. Analysis of covariance, with the pre-infusion or d0 concentration as a covariate, was used to compare differences between the placebo and Flu-IVIG treatment groups at d1 post-infusion. The p-value represents the difference between treatment groups for $\log_2$ d1 post-infusion controlling for pre-infusion (d1) IL-6 concentration. *** $P < 0.001$
